# Supplementary material for: Reduced cytochrome P-450 (CYP) 2D6 activity and Plasmodium vivax malaria risk in Amazonians: A retrospective, population-based cohort study
Source: PLoS Negl Trop Dis. 2026 Mar 27;20(3):e0014160. doi: 10.1371/journal.pntd.0014160 (PMC13048497; doi:10.1371/journal.pntd.0014160)
Supplement: S1 Fig — A, Location of the municipality of Mâncio Lima (black) in Acre State (gray) in the western part of Brazil (light gray), next to the border with Peru. B, Aerial photography of the municipality seat, the town of Mâncio Lima, taken by the first author. The map in panel A was created with QGIS software version 3.14, an open-source Geographic Information System (GIS) licensed under the GNU General Public License (https://bit.ly/2BSPB2F). Publicly available shape files provided from the Brazilian Institute of Geography and Statistics (IBGE) website (https://bit.ly/34gMq0S). All geographical data are used under the Creative Commons Attribution License (CC BY 4.0). (PDF) [file pntd.0014160.s002.pdf]

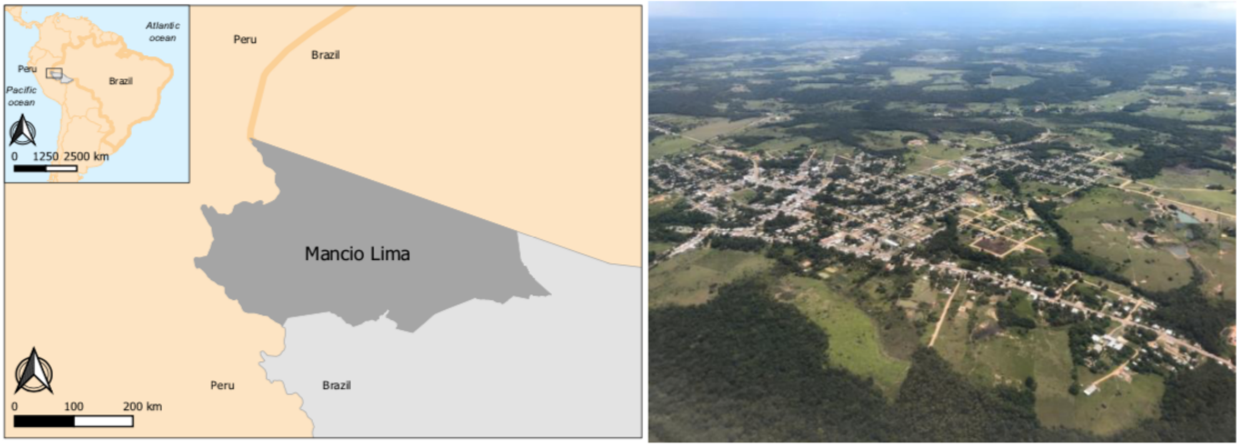

S1 Fig. Study site. Left panel, location of the municipality of Mâncio Lima (dark gray) in Acre State (light gray) in the western part of Brazil, along the border with Peru. Figure created with QGIS software version 3.14, an open-source Geographic Information System (GIS) licensed under the GNU General Public License (https://bit.ly/2BSPB2F). Publicly available shape files provided from the Brazilian Institute of Geography and Statistics (IBGE) website (https://bit.ly/34gMq0S). All geographical data are used under the Creative Commons Attribution License (CC BY 4.0). Right panel, aerial photography of the municipality seat, the town of Mâncio Lima, taken by the first author.
