# Supplementary material for: Reduced cytochrome P-450 (CYP) 2D6 activity and Plasmodium vivax malaria risk in Amazonians: A retrospective, population-based cohort study
Source: PLoS Negl Trop Dis. 2026 Mar 27;20(3):e0014160. doi: 10.1371/journal.pntd.0014160 (PMC13048497; doi:10.1371/journal.pntd.0014160)
Supplement: S2 Fig — Finger-prick blood samples were collected from 2,774 participants in the Mâncio Lima cohort study between April 2018 and November 2021. The Duffy blood group (FY) genotype was determined for 1,921 participants. Of those, one participant was excluded due to missing age information, 183 were excluded because they did not reside in the study area anytime between January 2014 and December 2018, 680 because complete CYP2D6 genotype information was not available, and 106 because they were Duffy (Fy)-negative. After all exclusions, 997 study participants remained in the retrospective cohort. (PDF) [file pntd.0014160.s003.pdf]

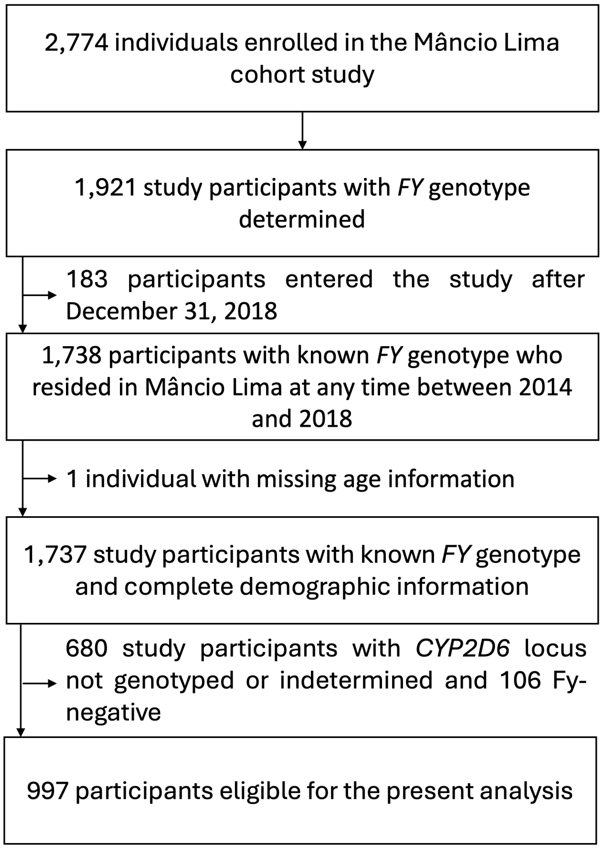

S2 Fig. Study flow diagram. Finger-prick blood samples were collected from 2,774 participants in the Mâncio Lima cohort study between April 2018 and November 2021. The Duffy blood group (*FY*) genotype was determined for 1,921 participants. Of those, one participant was excluded due to missing age information, 183 were excluded because they did not reside in the study site anytime between January 2014 and December 2018, 680 because they lacked complete *CYP2D6* genotype information, and 106 because they were Duffy (Fy)-negative. After all exclusions, 997 study participants were retained in the retrospective cohort.
