# Supplementary material for: Reduced cytochrome P-450 (CYP) 2D6 activity and Plasmodium vivax malaria risk in Amazonians: A retrospective, population-based cohort study
Source: PLoS Negl Trop Dis. 2026 Mar 27;20(3):e0014160. doi: 10.1371/journal.pntd.0014160 (PMC13048497; doi:10.1371/journal.pntd.0014160)
Supplement: S3 Fig — Malaria case records from 01 January 2014 through 31 December 2018 were retrieved from the SIVEP-Malaria database and matched to study participants. (PDF) [file pntd.0014160.s004.pdf]

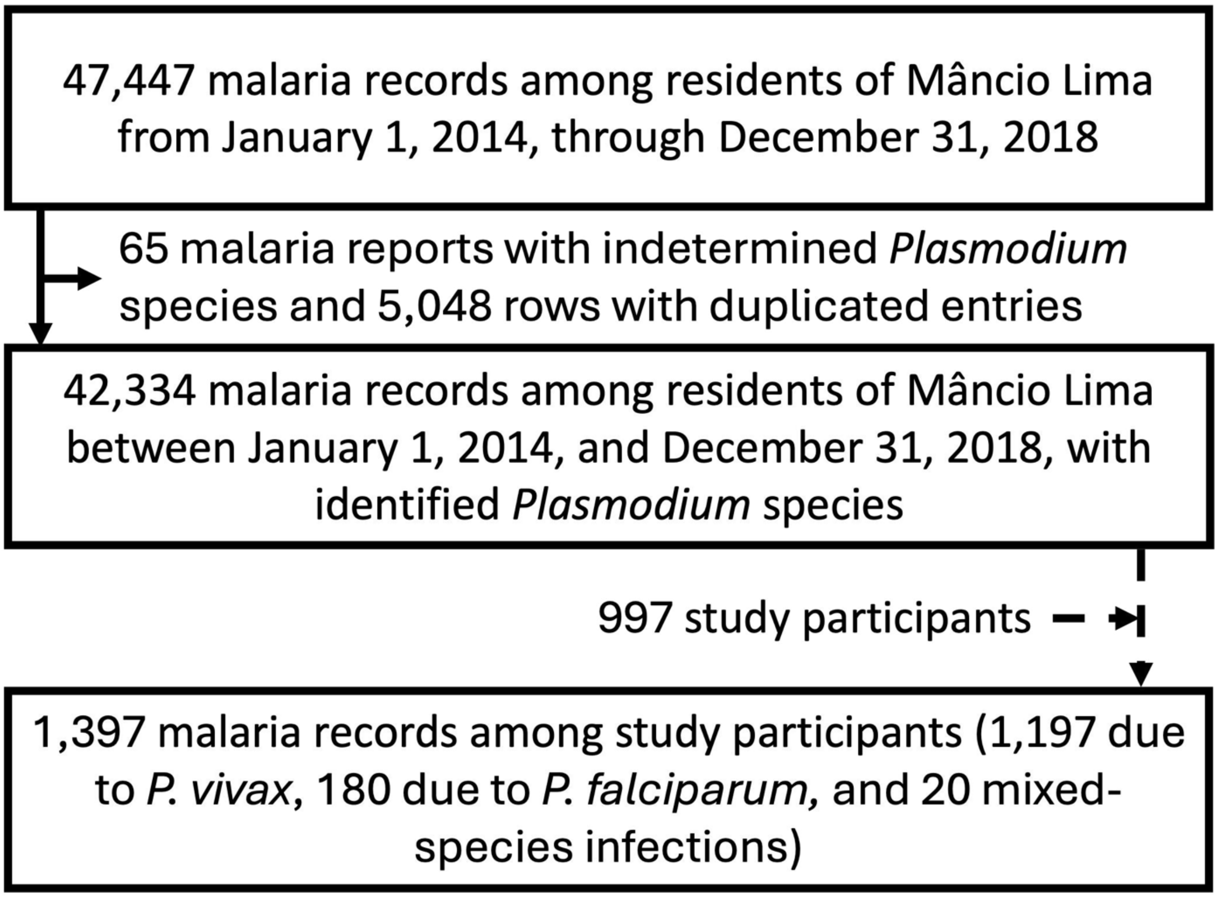

S3 Fig. Main study outcome. Malaria case records from 01 January 2014 through 31 December 2028 were retrieved from the SIVEP-Malaria database and matched to study participants.
