# Supplementary material for: Reduced cytochrome P-450 (CYP) 2D6 activity and Plasmodium vivax malaria risk in Amazonians: A retrospective, population-based cohort study
Source: PLoS Negl Trop Dis. 2026 Mar 27;20(3):e0014160. doi: 10.1371/journal.pntd.0014160 (PMC13048497; doi:10.1371/journal.pntd.0014160)
Supplement: S4 Fig — We identified the first or only symptomatic P. vivax infection experienced by 481 participants between 2014 and 2018 and excluded 15 episodes that were not treated with PQ or given a blood schizonticidal partner drug other than CQ. There were 466 participants in the time-to-recurrence analysis and 131 first or only P. vivax malaria recurrences were diagnosed during the 6-month follow-up period. (PDF) [file pntd.0014160.s005.pdf]

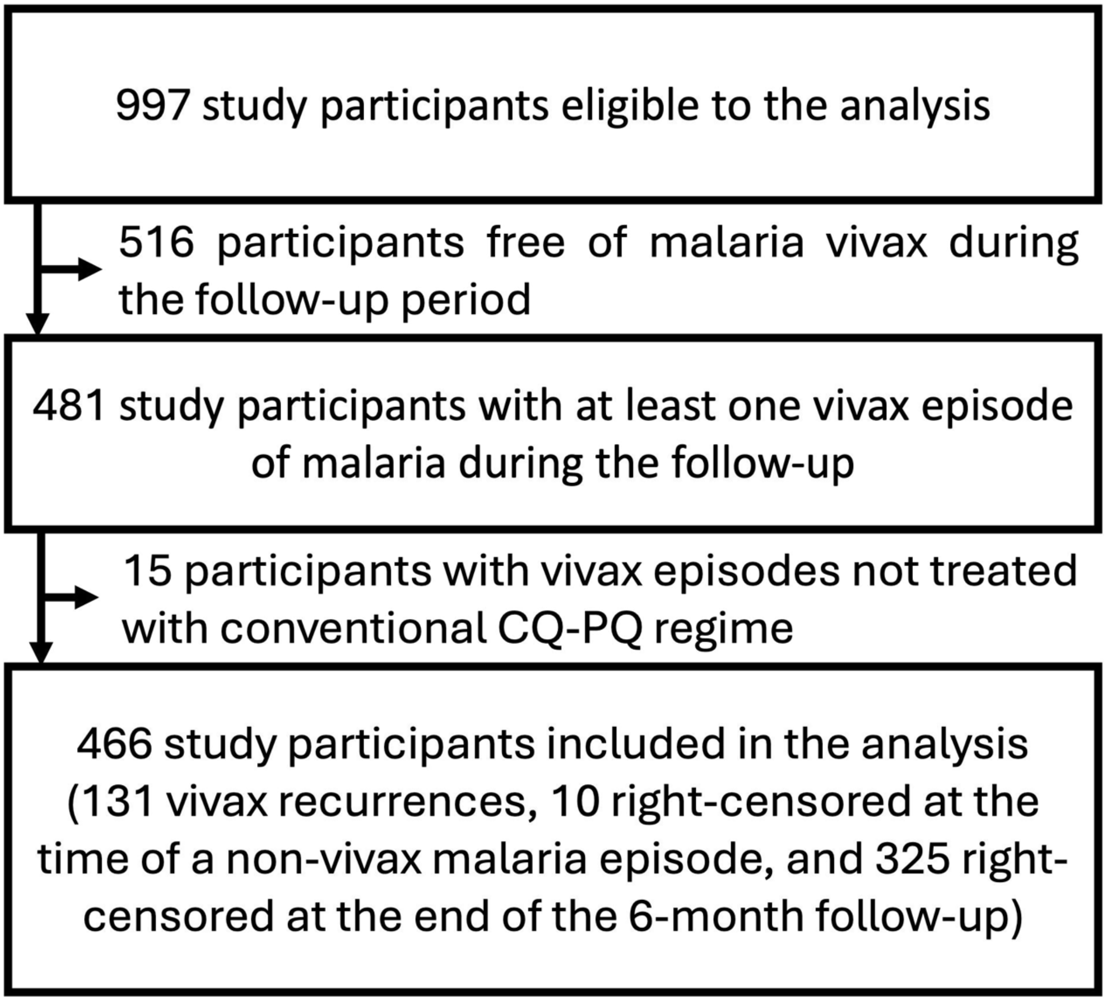

S4 Fig. Participants in the time-to-event analysis. We identified the first or only symptomatic *P. vivax* infection experienced by 481 participants between 2014 and 2018 and excluded 15 episodes that were not treated with PQ or given a blood schizonticidal partner drug other than CQ. There were 466 participants in the time-to-recurrence analysis and 131 first or only *P. vivax* malaria recurrences were diagnosed during the 6-month follow-up period.
