# Supplementary material for: Reduced cytochrome P-450 (CYP) 2D6 activity and Plasmodium vivax malaria risk in Amazonians: A retrospective, population-based cohort study
Source: PLoS Negl Trop Dis. 2026 Mar 27;20(3):e0014160. doi: 10.1371/journal.pntd.0014160 (PMC13048497; doi:10.1371/journal.pntd.0014160)
Supplement: S5 Fig — Case records were retrieved from the SIVEP-Malaria database and matched to study participants (n = 997). (PDF) [file pntd.0014160.s006.pdf]

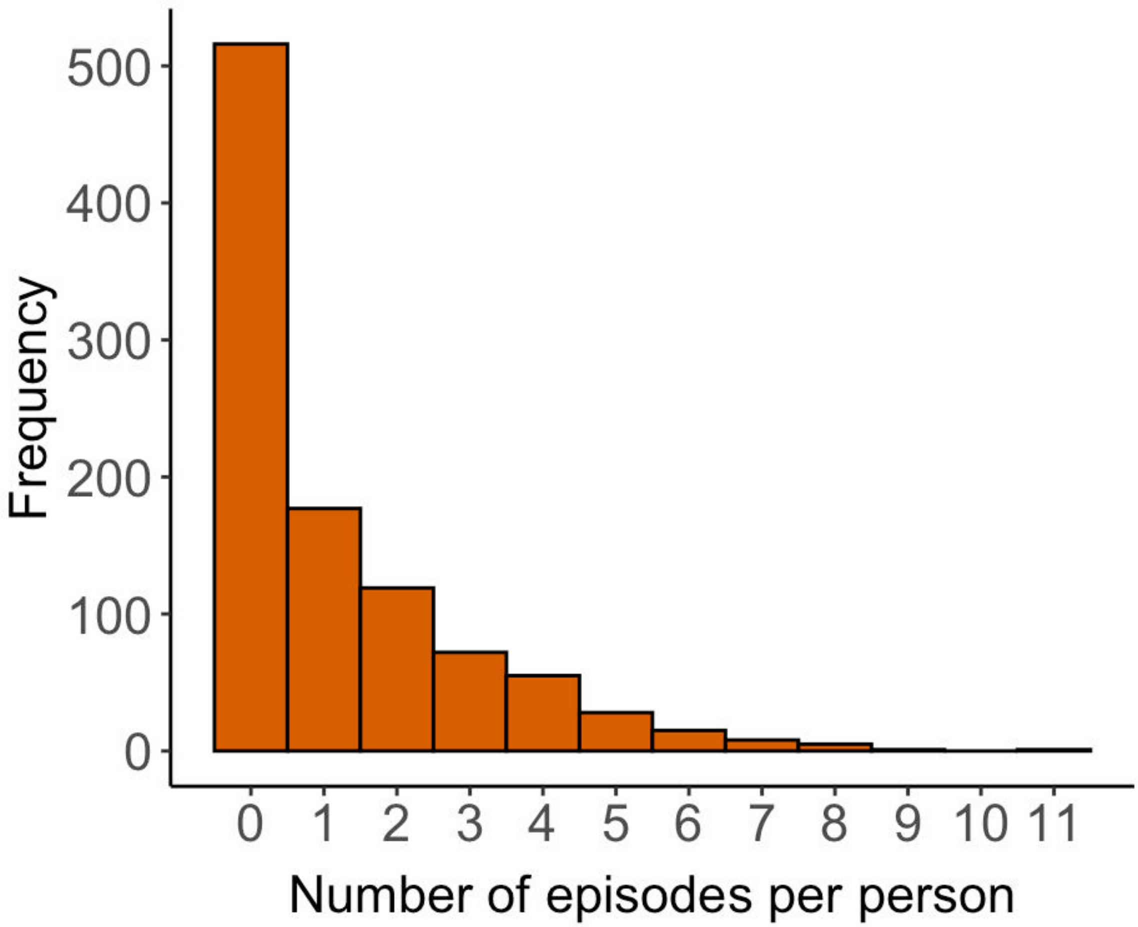

S5 Fig. Distribution of the number of *Plasmodium vivax* infections per study participant between January 1, 2014, December 31, 2028. Case records were retrieved from the SIVEP-Malaria database and matched to study participants (n = 997).
