# Supplementary material for: Reduced cytochrome P-450 (CYP) 2D6 activity and Plasmodium vivax malaria risk in Amazonians: A retrospective, population-based cohort study
Source: PLoS Negl Trop Dis. 2026 Mar 27;20(3):e0014160. doi: 10.1371/journal.pntd.0014160 (PMC13048497; doi:10.1371/journal.pntd.0014160)
Supplement: S1 Table — (PDF) [file pntd.0014160.s007.pdf]

S1 Table. Summary of published studies on CYP2D6 activity and risk of *Plasmodium vivax* malaria recurrence.

First author/year	Country(ies)	Treatment	Outcome	Low CYP2D6 activity threshold	Number with low vs. normal-high activity	Summary statistics and *P* value
St Jean 2016	Peru, Thailand, India	CQ + low-dose PQ	% with recurrence within 6 mo.	AS ≤1	8/23	OR = 9.18, *P* = 0.050
*Brasil 2018	Brazil	CQ + low-dose PQ	% with recurrence within 6 mo.	AS ≤1	37/153	RR = 1.89, *P* = 0.049
Brasil 2018	Brazil	CQ + low-dose PQ	Time to first recurrence within 6 mo.	AS ≤1	37/153	Not stated, *P* = 0.917
*Baird 2018	Indonesia	Artesunate, dihydroartemisinin-piperaquine, or artesunate-pyronaridine + high-dose PQ	% with recurrence within 12 mo.	Poor or intermediate metabolizer	30/37	OR = 7.52, *P* = 0.001
Baird 2018	Indonesia	Artesunate, dihydroartemisinin-piperaquine, or artesunate-pyronaridine + high-dose PQ	% with recurrence within 12 mo.	AS ≤1	32/35	OR = 9.45, *P* < 0.001
Baird 2018	Indonesia	Artesunate, dihydroartemisinin-piperaquine, or artesunate-pyronaridine + high-dose PQ	% with recurrence within 12 mo.	Log dextromethorphan-dextrorphan ratio ≥1.0	39/18	OR = 17.89, *P* < 0.001
Lacerda 2019	Brazil, Cambodia, Ethiopia, Peru, The Phillipines, Thailand	CQ + low-dose PQ	% with recurrence within 6 mo.	AS ≤1	36/80	OR = 1.68, *P* = 0.220
Ladeia-Andrade 2019	Brazil	CQ + low-dose PQ	Time to first recurrence within 6 mo.	AS ≤1	54/102	HR = 1.09, *P* = 0.782
*Silvino 2020	Brazil	CQ + low-dose PQ	Time to first recurrence within 6 mo.	AS ≤1	64/188	Not stated, *P* = 0.357
Silvino 2020	Brazil	CQ + low-dose PQ	Recurence incidence rate within 6 mo.	AS ≤1	64/188	IRR = 1.75, *P* = 0.003
Silvino 2020	Brazil	CQ + low-dose PQ	% with recurrence within 6 mo.	AS ≤1	64/188	OR = 1.94, *P* = 0.045
*Chamma-Siqueira 2022	Brazil	CQ + low-dose PQ supervised	% with recurrence within 24 wk.	AS ≤1	22/56	OR = 1.23, *P* = 0.679
Chamma-Siqueira 2022	Brazil	CQ + high-dose PQ supervised	% with recurrence within 24 wk.	AS ≤1	28/51	OR = 2.04, *P* = 0.252
Choi 2022	South Korea	Not stated	% with recurrence within unspecified time	AS ≤1	55/84	OR = 2.22, *P* = 0.019
Sutanto 2023	Indonesia	Dihydroartemisinin-piperaquine + high-dose PQ	% with recurrence within 6 mo.	No threshold; comparison across AS groups	20/30	Not stated, *P* = 0.421
*Salazar 2024	Brazil	CQ + low-dose PQ	% with recurrence within 6 mo.	AS ≤1	37/67	OR = 0.74, *P* = 0.514
Salazar 2024	Brazil	CQ + low-dose PQ	Time to first recurrence within 6 mo.	AS ≤1	37/67	Not stated, *P* = 0.423
Puça 2024	Brazil	CQ + low-dose PQ	% with single vs. multiple recurrences	AS ≤1	34/64	OR = 4.33, *P* = 0.001
Zeng 2025	Myanmar	CQ + low-dose PQ	% with recurrence within 12 mo.	AS ≤1.25	76/22	OR = 6.53, *P* < 0.001
*Da Silva 2025	Brazil	CQ + low-dose PQ	% with recurrence within 6 mo.	AS ≤1	11/50	OR = 3.80, *P* = 0.050
Da Silva 2025	Brazil	CQ + low-dose PQ	Time to first recurrence within 6 mo.	AS ≤1	11/50	Not stated, *P* = 0.301
*Sierra-Cifuentes 2025	Colombia	CQ + low-dose PQ	Time to first recurrence within 6 mo.	AS ≤1	13/58	HR = 1.45 (CI 95% 0.39-5.39)
Sierra-Cifuentes 2025	Colombia	CQ + low-dose PQ	% with recurrence within 6 mo.	AS ≤1	13/58	OR = 1.63, *P* = 0.511

Note: CQ = chloroquine, PQ = primaquine, AS = activity score, OR = odds ratio, RR = relative risk, HR = hazard ratio, IRR = incidence rate ratio, CI = confidence interval, mo = months, wk. = weeks. Low-dose PQ = 3.5 mg/kg over 7 or 14 days; high-dose PQ = 7.0 mg/kg over 14 days. Asterisks (*) indicate studies that included more than one population or were analyzed in different ways (see text).
