# Supplementary material for: Reduced cytochrome P-450 (CYP) 2D6 activity and Plasmodium vivax malaria risk in Amazonians: A retrospective, population-based cohort study
Source: PLoS Negl Trop Dis. 2026 Mar 27;20(3):e0014160. doi: 10.1371/journal.pntd.0014160 (PMC13048497; doi:10.1371/journal.pntd.0014160)
Supplement: S2 Table — (PDF) [file pntd.0014160.s008.pdf]

S2 Table Classical CYP2D6 phenotype classification and alternative classifications used in the present study.

CYP2D6 phenotype (number of participants within category)	CYP2D6 activity score (AS) range
**Classical AS classification (Clinical Pharmacogenetics Implementation Consortium), 4 groups**	
Poor metabolizer (PM) (*n* = 45)	0
Intermediate metabolizer (IM) (*n* = 234)	0.25 to 1.00
Normal metabolizer (NM) (*n* = 622)	1.25 to 2.25
Ultrarapid metabolizer (UM) (*n* = 96)	>2.25
**(A) Alternative AS categories, 2 groups**	
Null activity (*n* = 45)	0
Low, intermediate, normal, or high activity (*n* = 952)	≥0.25
**(B) Alternative AS categories, 2 groups**	
Null or low activity (*n* = 70)	0 to 0.25
Intermediate, normal, or high activity (*n* = 927)	≥0.50
**(C) Alternative AS categories, 2 groups**	
Null or low activity (*n* = 85)	0 to 0.50
Intermediate, normal or high activity (*n* = 912)	≥0.75
**(D) Alternative AS categories, 2 groups**	
Null, low, or intermediate activity (*n* = 92)	0 to 0.75
Normal or high activity (*n* = 905)	≥ 1.0
**(E) Alternative AS categories, 2 groups**	
Null, low, or intermediate activity (*n* = 279)	0 to 1.00
Normal, or high activity (*n* = 718)	≥ 1.25
**(F) Alternative AS categories, 2 groups**	
Null, low, or intermediate activity (*n* = 405)	0 to 1.25
Normal or high activity (*n* = 592)	≥ 1.50
**(G) Alternative AS categories, 3 groups**	
Null or low activity (*n* = 70)	0 to 0.25
Intermediate activity (*n* = 209)	0.50 to 1.00
Normal or high activity (*n* = 718)	≥ 1.25
