# Supplementary material for: Reduced cytochrome P-450 (CYP) 2D6 activity and Plasmodium vivax malaria risk in Amazonians: A retrospective, population-based cohort study
Source: PLoS Negl Trop Dis. 2026 Mar 27;20(3):e0014160. doi: 10.1371/journal.pntd.0014160 (PMC13048497; doi:10.1371/journal.pntd.0014160)
Supplement: S3 Table — (PDF) [file pntd.0014160.s009.pdf]

S3 Table. Characteristics of Duffy (Fy)-positive Mâncio Lima cohort participants included in the present analysis (*n* = 997) and those excluded from the present analysis due to missing CYP2D6 genotype information (*n* = 634).

	**Fy-positive participants in the Mâncio Lima cohort study**		
**Characteristic**	**Included in the analysis (*n* = 997)**	**Excluded from the analysis (*n* = 634)**	***P* value**
**Sex**			0.319
Female	525 (52.66%)	317 (50.00%)	
Male	472 (47.34%)	317 (50.00%)	
**Mean age, years (SD)**	29.06 (SD = 19.88)	29.60 (SD = 20.20)	0.602
**Wealth index tercile**			0.153
1 (poorest)	347 (34.80%)	209 (32.97%)	
2	348 (34.90%)	204 (32.18%)	
3 (wealthiest)	302 (30.29%)	221 (34.86%)	
**Duffy (*FY*) genotype**			0.982
*FY*01/FY*01N.01*	173 (17.35%)	112 (17.67%)	
*FY*01/FY*01*	155 (15.55%)	96 (15.14%)	
*FY*02/FY*01N.01*	172 (17.25%)	107 (16.88%)	
*FY*02/FY*02*	170 (17.05%)	115 (18.14%)	
*FY*01/FY*02*	327 (32.80%)	204 (32.18%)	

Note: SD = standard deviation
