# Supplementary material for: Reduced cytochrome P-450 (CYP) 2D6 activity and Plasmodium vivax malaria risk in Amazonians: A retrospective, population-based cohort study
Source: PLoS Negl Trop Dis. 2026 Mar 27;20(3):e0014160. doi: 10.1371/journal.pntd.0014160 (PMC13048497; doi:10.1371/journal.pntd.0014160)
Supplement: S4 Table — (PDF) [file pntd.0014160.s010.pdf]

S4 Table. Distribution of cytochrome P-450 (CYP) 2D6 activity scores predicted from genotypes among 997 Mâncio Lima cohort participants, 2014–18.

Activity Score	CPIC Classification	No. of participants	Frequency, % (95% CI)	Cumulative frequency, % (95% CI)
0.00	Poor metabolizer	45	4.5 (3.4, 6.0)	4.5 (3.4, 6.0)
0.25	Intermediate metabolizer	25	2.5 (1.7, 3.7)	7.0 (5.6, 8.8)
0.50	Intermediate metabolizer	15	1.5 (0.9, 2.5)	8.5 (6.9, 10.4)
0.75	Intermediate metabolizer	7	0.7 (0.3, 1.4)	9.2 (7.6, 11.2)
1.0	Intermediate metabolizer	187	18.8 (16.5, 21.3)	28.0 (25.3, 30.9)
1.25	Normal metabolizer	126	12.6 (10.7, 14.8)	40.6 (37.6, 43.7)
1.50	Normal metabolizer	36	3.6 (2.6, 5.0)	44.2 (41.2, 47.3)
2.00	Normal metabolizer	460	46.1 (43.1, 49.2)	90.4 (88.4, 92.1)
>2.25	Ultrarapid metabolizer	96	9.6 (7.9, 11.6)	100.0 (99.6, 100.0)

CPIC = Clinical Pharmacogenetics Implementation Consortium [6]; CI = confidence interval
