# Supplementary material for: Reduced cytochrome P-450 (CYP) 2D6 activity and Plasmodium vivax malaria risk in Amazonians: A retrospective, population-based cohort study
Source: PLoS Negl Trop Dis. 2026 Mar 27;20(3):e0014160. doi: 10.1371/journal.pntd.0014160 (PMC13048497; doi:10.1371/journal.pntd.0014160)
Supplement: S5 Table — (PDF) [file pntd.0014160.s011.pdf]

S5 Table. Association between different activity score thresholds to define "low" CYP2D6 activity and the risk of *P. vivax* malaria recurrence within 6 months of CQ-PQ treatment, as estimated by logistic regression analysis, among 466 Mâncio Lima cohort participants, 2014–18.

AS threshold	No. of participants in the "low-activity" group	Odds Ratio (95% confidence interval)	*P* value
0.00	45	2.48 (0.48, 10.78)	0.254
≤ 0.25	70	3.98 (1.28, 11.84)	0.014
≤ 0.50	85	3.42 (1.13, 9.80)	0.025
≤ 0.75	92	3.06 (1.04, 8.53)	0.036
≤ 1.00	279	1.65 (0.78, 3.51)	0.191
≤ 1.25	405	0.92 (0.46, 1.83)	0.814

Note: Logistic regression models were used to estimate odds ratios and 95% confidence intervals for the association between CYP2D6 activity levels and risk of presenting one of more recurrences at the end of the 6–month follow-up, while adjusting for sex, age, *FY* genotype, wealth index, and individual's time at risk.
