# Supplementary material for: Reduced cytochrome P-450 (CYP) 2D6 activity and Plasmodium vivax malaria risk in Amazonians: A retrospective, population-based cohort study
Source: PLoS Negl Trop Dis. 2026 Mar 27;20(3):e0014160. doi: 10.1371/journal.pntd.0014160 (PMC13048497; doi:10.1371/journal.pntd.0014160)
Supplement: S1 File — (DOCX) [file pntd.0014160.s012.docx]

**S1 File**

**Reduced cytochrome P-450 (CYP) 2D6 activity and *Plasmodium vivax* malaria risk in Amazonians: a retrospective, population-based cohort study**

**1. Literature review**

We used the following search terms to identify publications describing previous observational studies and clinical trials, in English, Spanish, Portuguese, or French, that addressed the impact of CYP2D6 activity on *P. vivax* malaria relapse risk following treatment with primaquine (PQ) and a blood schizonticide: ((Plasmodium AND vivax) OR (vivax malaria)) AND primaquine AND (CYP2D6 OR cytochrome P450 D6). We identified 55 articles that were included in the PubMed and SciELO databases. We removed duplicates, reviews, case reports and case series, studies that did not classify patients according to activity scores (AS), and off-topic publications. Fourteen studies comparing recurrence rates or time-to-recurrence in PQ-treated *P. vivax* malaria patients across CYP2D6 activity levels were retained for analysis (S1 Table).

Twelve studies defined low CYP2D6 activity as AS ≤1, implying that poor and intermediate metabolizers were grouped together as “low metabolic activity” [1]. One study used AS ≤1.25 as the cut-off value, implying that some normal metabolizers (AS = 1.25) were grouped together with poor (AS = 0) and intermediate metabolizers (AS between 0.25 and 1.0) [2]. Another study used no cut-off values to define CYP2D6 activity [3]. Only one study determined the CYP2D6 activity phenotype by measuring the dextromethorphan–dextrorphan ratio following the oral administration of dextromethorphan hydrobromide, instead of inferring phenotypes from genotypes [4].

PQ doses used across studies ranged between 3.5 mg/kg over 7 or 14 days (low dose) and 7.0 mg/kg over 14 days (high dose) and were given either concomitantly with, or up to 28 after, the administration of blood schizonticides. Twelve studies used chloroquine (CQ; 25 mg/kg over 3 days) as the blood schizonticidal partner, while two clinical trials from Indonesia used artesunate alone, dihydroartemisinin–piperaquine, or artesunate–pyronaridine as blood schizonticides [3,4].

Data from the studies marked with an asterisk in S1 Table were analyzed in different ways, either by using different cut-off values to define low CYP2D6 activity [4] or by combining time-to-recurrence analysis with comparisons of the proportion of patients experiencing recurrences by the end of the follow-up [5-8]. Data from one study were analyzed separately for low and high PQ doses [9].

Different CYP2D6 genotyping strategies were used across studies and the number of CYP2D6 variants (single-nucleotide polymorphisms and deletions) analyzed ranged from 7 to 12. Not all studies reported an analysis of *CYP2D6* copy number variation. Importantly, CYP2D6 activity may have been misclassified if gene variants and duplications were missed by the genotyping strategy; when no polymorphism is found, a normal-function *CYP2D6*1* allele is the default assignment. Rare variants are usually missed [10]. Comparisons across studies may also be limited because the reduced-function *CYP2D6*10* allele, which is particularly common among East Asians [11,12], has been assigned an activity value of either 0.5 or 0.25 over time. This allele was recently downgraded to 0.25 [1], but only the latest studies have adopted this new value.

**2. CYP2D6 activity and the anti-relapse efficacy of tafenoquine**

Alternative anti-relapse regimens are urgently needed for the radical cure of *P. vivax* malaria in patients with low CYP2D6 activity. Tafenoquine (TQ), a newly available 8-aminoquinoline analog that kills all malaria parasite stages, including *P. vivax* hypnozoites [13], might be a valuable option. However, the mechanism of action of TQ remains poorly understood and whether CYP2D6-dependent activation is required for its therapeutic activity is unclear [14]. Data from a knock-out mouse model suggest that CYP2D6-dependent biotransformation is required for TQ effect against hepatic stages of malaria parasites [15], but not necessarily schizonts and gametocytes [16]. Conflicting results between these experimental studies may be partially due to differences in the TQ doses used, approximately 8 times higher in Milner *et al*. (2016) [16] compared with Marcsisin *et al*. (2014) [15]. Data from clinical studies are very limited; few low-activity patients were enrolled in clinical trials of TQ and its anti-relapse efficacy did not appear to be significantly reduced among patients with intermediate or poor CYP2D6 activity [17].

**3. Study site and population**

Located in the upper Juruá Valley region of Acre State (S1 Fig.), the municipality of Mâncio Lima is characterized by an equatorial humid climate with most rainfall between November and April. There is year-long malaria transmission, with *Anopheles* (*Nyssorhynchus*) *darlingi* serving as the primary vector and more than 80% of the local cases being due to *P. vivax*.

S2 Fig. summarizes the steps for retrospective cohort construction. Briefly, we included study participants with known Duffy blood group (*FY*) and *CYP2D6* genotypes who resided in Mâncio Lima anytime between 2014 and 2018. We excluded Duffy-negative participants carrying the T-67C nucleotide substitution in the globin transcription factor–1 (GATA–1) binding motif of the *ACKR1* gene. The date of entry in the retrospective cohort was the date of birth, the date the participant moved to Mâncio Lima, for those not enumerated in the 2015–16 census, or January 1, 2014 – whichever was the most recent.

**4. Malaria treatment**

Free treatment with regimens recommended by the Ministry of Health of Brazil [18] is widely available for laboratory-confirmed malaria in the study site. *P. vivax* infections are routinely treated with chloroquine (CQ; 25 mg/kg over 3 days) and low-dose primaquine (PQ; 3.5 mg/kg over 7 or 14 days) [19,20]. High-dose PQ (7.0 mg/kg over 14 days) is superior to low-dose PQ in preventing *P. vivax* malaria recurrences in our study area [9], but is not currently recommended by the Ministry of Health of Brazil [18]. The recommendation of treating PQ-ineligible patients with a weekly prophylactic CQ dose of 300 mg over 12 weeks following CQ-only treatment [18] is rarely adhered to. *P. falciparum* infections are treated with a 3-day course of either artemether (2 to 4 mg/kg/day) plus lumefantrine (12 to 24 mg/kg/day) [21] or artesunate (4 mg/kg/day) plus mefloquine (8 mg/kg/day) [22], followed by a single dose of 0.75 mg/kg PQ, for gametocyte clearance [18]. Point-of-care screening for glucose-6-phosphate dehydrogenase (G6PD) deficiency is not routinely carried out prior to PQ administration in Brazil [18], but is currently being implemented for TQ administration.

**5.** ***CYP2D6* genotyping assays**

Methods for *CYP2D6* genotyping have been described in detail elsewhere [23]. Briefly, template DNA was isolated from 50–µL capillary blood samples using the DNA Investigator kit (Qiagen, Hilden, Germany) on an automated QIASymphony platform (Qiagen). Prior to *CYP2D6* genotyping, we carried out a DNA pre-amplification step on a Veriti thermal cycler (Thermo Fisher Scientific, Waltham, MA) with the following cycling program: 95°C for 10 minutes, followed by 14 cycles of 95°C for 15 seconds, 60°C for 4 minutes, and 100°C for 10 minutes. The final reaction volume of 5 μL contained 2.5 μL of TaqMan PreAmp MasterMix, 1.25 μL of OpenArray PreAmp Pool with *CYP2D6*-specific primers, and 1.25 μL of genomic DNA.

We used custom OpenArray assays (Thermo Fisher Scientific) with specific primers and probes to identify nine single-nucleotide polymorphisms (G-1584C [rs1080985], G31A [rs769258], C100T [rs1065852], C1023T [rs28371706], G1846A [rs3892097], G2850A [rs16947], G2988A [rs28371725], G3183A [rs59421388], and G4180C [rs1135840]) and three deletions (1707delT [rs5030655], 2549delA [rs35742686], and 2613_2615delAGA [rs5030656]) at the *CYP2D6* locus*.* We run genotyping reactions on OpenArray microplates containing 3,072 through-holes, with each 3.5-μL reaction containing 2 μL of TaqMan OpenArray Genotyping Master Mix (Thermo Fisher Scientific) and 1.5 μL of the pre-amplification product (corresponding to 40 ng/μL of DNA). The thermal cycling protocol recommended by the manufacturer was run on a QuantStudio 12K Flex Real-Time PCR System (Thermo Fisher Scientific).

We used the Hs00010001_cn assay (Thermo Fisher Scientific), which targets exon 9, to estimate the number of *CYP2D6* gene copies. Samples were tested as described [6], using the human *RNase P* gene as reference. We used 384-well microplates with 10-μL reaction volumes per well containing 10 ng/μL of template DNA. Samples were run in triplicate on a ViiA7 Real-Time PCR System (Thermo Fisher Scientific) with the following cycling program: 95°C for 10 minutes, followed by 40 cycles of 95°C for 15 seconds and 60°C for 60 seconds. Results were analyzed using the CopyCaller software version 2.0 (Thermo Fisher Scientific).

**6. Estimating activity scores (AS)**

We inferred CYP2D6 haplotypes using the Haplo2D6 web tool (https://bioinfo.dcc.ufmg.br/Haplo2D6/), which integrates the PHASE software version 2.1 [24], with 50,000 burn-in steps followed by 400,000 iterations, and a thinning interval of 1,000 iterations. Haplo2D6 allows for automated haplotype assignment, activity score calculation, and phenotype inference. Metabolic activity values were assigned to *CYP2D6* alleles as described: an activity value of 1 was assigned to the fully functional *CYP2D6*1* allele, while 0 was assigned to non-functional alleles, and values between 0.25 and 0.5 were assigned to reduced-activity alleles defined according to the Pharmacogene Variation Consortium guidelines (PharmVar) (<https://www.pharmvar.org/gene/CYP2D6>) [1]. The activity value was multiplied when *CYP2D6* allele duplications/multiplications were present. Activity values assigned to individual *CYP2D6* alleles were summed to obtain activity scores (AS) in the diploid genome.

**7. Inferring phenotypes from *CYP2D6* genotypes**

Based on AS, individuals have been usually classified as poor metabolizers (PM; AS = 0), intermediate metabolizers (IM; AS between 0.25 and 1), normal metabolizers (NM; AS between 1.25 and 2.25) or ultrarapid metabolizers (UM; AS greater than 2.25) (S2 Table). However, this grouping may not reveal potentially important phenotypic differences among individuals. For instance, CYP2D6 activity varies widely within the large group of “intermediate metabolizers” (*n* = 234 individuals in our study) and this group includes individuals with both very low (AS of 0.25) and half-normal (AS = 1) activity [1]. Moreover, although individuals with AS of 1.25 (*n* = 126 in our study) have often been classified as normal metabolizers, they appear be at increased risk of relapses following PQ treatment in Myanmar [2].

Here, we explored alternative ways of classifying study participants according to inferred CYP2D6 phenotype (A through G, S2 Table). Of note, alternative E (two groups, AS ≤1 vs. AS > 1) is the most commonly way of dichotomizing AS in studies looking for an association between CYP2D6 activity and risk of vivax malaria recurrence (S1 Table), while alternative F (two groups, AS ≤1.25 vs. AS > 1.25) was adopted in the recent study by Zeng *et al*. (2025) [2].

**8. Duffy (*FY*) blood group genotyping**

We genotyped two single-nucleotide polymorphisms at the *FY* locus. The first is the -67T→C substitution in the RBC-specific globin transcription factor–1 (GATA–1) binding motif (rs2814778), which suppresses Fy expression on the erythrocyte surface. We excluded from further analysis all participants homozygous for the *FY*01N.01* allele, corresponding to the Fy(a-b-) or Fy-negative (null) phenotype. The second polymorphism is the 125G→A substitution in exon 2 (rs12075), which differentiates the *FY*01* and *FY*02* alleles associated with the Fy(a+), Fy(b+), Fy(a+b+) phenotypes. All Fy-positive participants were included in our analyses, but *FY* genotype was included as a potential confounder because the Fy(a+), Fy(b+), Fy(a+b+) phenotypes have been associated with differential susceptibility to *P. vivax* infection in Amazonians [25].

We used TaqMan assays (C__15769614_10 and C 2493442_20) for *FY* genotyping, with hydrolysis probes labelled with the VIC and FAM fluorochromes (ThermoFisher Scientific, Waltham, MA). The PCR amplification was carried out on a ViiA7 Real-Time PCR System (ThermoFisher Scientific) essentially as described [26] with one cycle of polymerase activation at 95°C for 10 minutes, followed by 50 cycles of denaturation at 95°C for 15 seconds and annealing/extension at 60°C for 1 minute.

**9. Malaria case finding**

We searched the SIVEP-Malaria database of malaria case notifications [27] for cases diagnosed by thick-smear microscopy or rapid diagnostic test, irrespective of parasite density and presence of symptoms, in 997 Fy-positive individuals with known *CYP2D6* genotype participating in the Mâncio Lima Cohort Study who resided in the study site anytime from January 1, 2014, through December 31, 2018 (Supplementary Figure 3). We assume that nearly all malaria cases diagnosed in the study population could be retrieved, because malaria is a notifiable disease in Brazil and diagnostic testing and antimalarial medications are not available outside the public sector [28].

We excluded records with “non-falciparum” results obtained with rapid diagnostic tests with the P.f/Pan format (*n* = 65). Such tests, which detect the histidine-rich protein 2 (HRP2) antigen of *P. falciparum* and a common, genus-specific lactate dehydrogenase (pLDH) of *Plasmodium* species, may miss falciparum malaria due to the high (46.5%) frequency of *HRP2* gene deletion in local *P. falciparum* populations [29]. Therefore, “non-falciparum” results may be obtained for infections with either *P. falciparum* (with HRP2 deletion) or *P. vivax* and were considered “undetermined species” (S3 Fig.). However, results from rapid diagnostic tests with the P.f/P.v. format, which include separate bands with species-specific antibodies for the detection of pLDH from *P. falciparum* and *P. vivax,* were computed. We also excluded duplicated entries with the same patient’s name, patient’s mother’s name and date of malaria testing.

**10. Record linkage**

Record linkage strategies have been described in detail elsewhere [30]. Briefly, we capitalized all names (individual and mother’s name) in SIVEP-Malaria entries and the Mâncio Lima Cohort Study database and removed accents of accentuated letters and UTF-8 characters that are not *latin-1* (to avoid non-textual characters). For records with missing date of birth, we inferred the birth date as January 1st of the year corresponding to the difference between the year of examination and the self-reported participant’s age.

To determine whether a SIVEP-Malaria entry $i$ could be linked to a study participant $j$, we searched the database for case records that matched the patient’s name, the patient’s mother’s name, sex, age, and date of birth. We first computed the Jaro-Winkler distance [31], using 0.05 as a prefix scale, to assess the similarity between names. Specifically, we calculated the distances $dₙ(i, j)$ and $dₘ(i, j)$ between the name and mother’s name, respectively, between SIVEP entry $i$ and study participant $j$. A SIVEP-Malaria entry was associated with one or more study participants if it had the same sex, year of birth (of ±1 year) and satisfied the 3 conditions below:

1. $dₙ\left( i, j \right)\leq\tau$;
2. $dₘ(i, j)\leq\tau$;
3. $d\left( i, j \right)=1-\left( 1-dₙ\left( i, j \right) \right)\left( 1-dₘ\left( i, j \right) \right)\leq\tau;$

where $\tau$ is a predefined threshold distance for name similarity (see below). If the mother’s name of a SIVEP-Malaria entry $i$ or a study participant $j$ was missing, conditions 1-3 were replaced with $dₙ\left( i, j \right)<0.1$ [32]. If the participant’s name and mother’s name were both available, conditions 1-3 could also be replaced by an identical phonetic representation of the name and mother’s name between SIVEP entry $i$ and study participant $j$, as implemented in the package *SoundexBR* [33]. If SIVEP entry $i$ was associated with multiple study participants, we chose the participant with closest name (i.e., the participant $j$ with minimum $dₙ\left( i, j \right)$).

To define the optimal value of $\tau$, we applied our matching algorithm to a subset of SIVEP-Malaria entries from October 1, 2015, to September 30, 2016. The algorithm returned the most likely match (if any) for every SIVEP-Malaria entry. We manually verified whether each most likely match was a true correspondence. To define the threshold $\tau$, we maximized the reward function

$$R\left( \tau\right)= \mathrm{TPR}\left( \tau\right)+ \lambda\cdot\mathrm{TNR}\left( \tau\right),$$

where $\lambda$ is a constant that controls the rate between false positives and false negatives. We chose $\lambda= 5$ to penalize more a false positive than a false negative, resulting in a threshold $\tau_{opt} = 0.2668$.

We next applied an interval equal or greater than 28 days between two or more consecutive cases to count the latter episode as a new malaria infection. When different species were detected in samples obtained less than 28 days apart, the participant was considered to have a single episode of mixed-species infection. Finally, we excluded 53 malaria case records that occurred at a time the participant did not live in the study site. This resulted in 1,397 malaria records among study participants (1,197 due to *P. vivax*, 180 due to *P. falciparum* and 20 mixed-species infections) (S3 Fig.). Only single-species *P. vivax* infections were further considered in this study.

**11. Comparison between study participants and non-participants**

Participants in the retrospective Mâncio Lima cohort study (n = 997) did not differ significantly, in terms of age, sex, *FY* genotype, and wealth index, from the 634 Fy-positive participants who were excluded due to missing CYP2D6 genotype information (S3 Table).

**12. Timing of *Plasmodium vivax* relapses**

Studies of vivax malaria cases imported from the Amazon to non-endemic areas of Brazil suggest over 90% of the relapses occur within the first 6 months after the primary infection [34]; 71.6% of them occur between days 30 and 120 [35]. The median time to the first relapse has been estimated at 108 days [36], roughly consistent with a model-based pooled estimate of 65 days (95% confidence interval, 18-113 days) for *P. vivax* infections in South America [37]. Based on these data, we defined 180 days as the follow-up duration required to maximise the probability of capturing relapses in our time-to-event analysis. A longer follow-up would marginally increase sensitivity (few additional relapses included) at the expense of lower specificity (more reinfections included).

**13. CYP2D6 activity and time to *Plasmodium vivax* recurrence**

The distribution of CYP 2D6 activity scores predicted from genotypes among 997 Mâncio Lima cohort participants is shown in S4 Table. Table 1 (main text) shows the results of separate Cox regression models that estimated hazard ratios (HRs), with 95% confidence intervals (CIs), for the association between time-to-first-recurrence and CYP2D6 activity level, while adjusting for sex, age at the time of diagnosis (0–16, 17–40, and >40 years), *FY* genotype (*FY*01/FY*01, FY*01/FY*01N.01*, *FY*02/FY*02, FY*02/FY*01N.01,* or *FY*01/FY**02; International Society of Blood Transfusion – <https://www.isbtweb.org/resource/008fy.html>), and wealth index tercile. In this exploratory analysis, we used 6 different AS thresholds ranging from 0 to 1.25 to define reduced CYP2D6 activity (alternative classifications A through F in S2 Table).

The time-to-event analysis comprised 466 people who experienced at least one *P. vivax* infection registered in the SIVEP-Malaria database who were treated with CQ-PQ between January 2014 and December 2018; the endpoint was the first *P. vivax* recurrence diagnosed between days 28 and 180 after starting CQ–PQ treatment (*n* = 131 events). Survival times of patients who remained free of *P. vivax* malaria recurrence were right-censored at the end of the 180-day follow-up, at the time the patient left the study site, at the time the patient experienced a non-vivax malaria episode prior to any *P. vivax* recurrence, or on December 31, 2018, whatever came first. We used the Schoenfeld residuals test to confirm that the proportional-hazards assumption has not been violated.

For comparability with previous studies (S1 Table), we also compared recurrence rates within 6 months of CQ-PQ treatment, among patients with different CYP2D6 activity levels, using the same AS thresholds as above. To this end, we used logistic regression analysis to estimate odds ratios (ORs) and their 95% CIs for the association between CYP2D6 activity and *P. vivax* recurrence, while adjusting for sex, age, *FY* genotype, and socioeconomic status. Because participants differed in follow-up duration, the log-transformed individual’s time at risk (in days) was added as a covariate. Results are shown in S5 Table.

We next compared the time-to-recurrence across three CYP2D6 activity levels: null or very low (AS ≤ 0.25), intermediate (AS between 0.5 and 1.0), and normal/high (AS > 1.0). Table 2 (main text) shows the results of the Cox regression model adjusted for sex, age at the time of diagnosis, *FY* genotype, and wealth index tercile as above. The Kaplan-Meier survival curves are shown in Figure 1 (main text).

**14. CYP2D6 activity and *Plasmodium vivax* malaria incidence**

The overall incidence of *P. vivax* malaria over 5 years among all 997 study participants, due to either relapses or new infections, did not differ significantly according to CYP2D6 activity in our cohort population (Table 4, main text). However, once infected symptomatically, study participants with null/low CYP2D6 activity had more than twice the risk of recurrence compared to those with normal/high CYP2D6 activity (Table 2, main text). We offer a possible explanation for this paradoxical finding.

An age-stratified analysis of incidence rates shows a higher incidence rate among the 29 children and adolescents aged 0–16 years with null/low CYP2D6 activity (34.87 cases/100 person-years; 95% confidence interval [CI], 25.02 to 47.30), compared to 61 people of the same age with intermediate CYP2D6 activity (18.34 cases/100 person-years; 95% CI, 13.38 to 24.55) and 233 with normal/high CYP2D6 activity (23.13 cases/100 person-years; 95% CI, 20.23 to 26.32). Such a difference is not observed in the older age groups (Table 3, main text). Of note, among participants with null/low CYP2D6 activity, those aged 0–16 years had a greater incidence of vivax malaria than young adults aged 17–40 years (34.87 *vs* 27.17 cases/100 person-years). The opposite trend is observed among participants with intermediate and normal/high CYP2D6 activity, with a greater incidence in young adults who are typically more exposed to malaria due to occupational and behavioral characteristics [38].

We argue that clinical relapses (i.e., symptomatic infections originating from hypnozoites) may contribute substantially to the higher incidence of vivax malaria observed in naïve children with null/low CYP2D6 activity. Importantly, repeated *P. vivax* infections, either relapses or new infections, are associated with an increased risk of anemia and other adverse impacts on children’s health [39,40]. Adults with null/low CYP2D6 activity, however, may have developed some degree of immunity to blood-stage parasites after experiencing repeated relapses since birth. They are more likely to have acquired some degree of partial immunity that suppresses the clinical manifestations of relapses and new infections, therefore decreasing the apparent burden of clinical malaria captured by our retrospective cohort study. By contrast, participants with intermediate or normal/high CYP2D6 activity remain susceptible to clinical vivax malaria until well into adulthood, when exposure to infection is greatest [38].

Our hypothesis implies that the impact of CYP2D6 activity on the frequency of relapse is modified by age- and exposure-dependent acquired immunity [6,23]. Our failure to detect a statistically significant (at the 5% level) effect modification by age in the association between reduced CYP2D6 activity and vivax malaria risk (Table 4, main text) may be due to lack of statistical power, given the small number of participants in the highest risk group (children and adolescents with AS ≤ 0.25; *n* = 29) and lower overall incidence of vivax malaria in this age group compared with young adults (23.28 *vs* 31.47 cases/100 person-years).

Differences in CYP2D6 activity appear to contribute little to the marked individual variation in vivax malaria risk seen in the general population of Mâncio Lima [38]. We suggest that the excess of malaria cases due to symptomatic relapses, among participants aged 0-16 years with AS ≤ 0.25, adds relatively little to the community-wide burden of vivax malaria, all ages considered. Semi-immune adults with null/low CYP2D6 activity may continue to experience frequent relapses, but an increasingly higher proportion of them are submicroscopic and asymptomatic with increasing age and immunity [41] and can easily be missed by routine surveillance.

In summary, we argue that symptomatic relapses may account for a relatively small fraction of the total burden of repeated vivax malaria episodes detected by routine surveillance in general population, although asymptomatic and submicroscopic relapses may remain frequent among semi-immune adults with severely reduced CYP2D6 activity.

**S1 File References**

1. Caudle KE, Sangkuhl K, Whirl-Carrillo M, Swen JJ, Haidar CE, Klein TE, Gammal RS, Relling MV, Scott SA, Hertz DL, Guchelaar HJ, Gaedigk A. Standardizing CYP2D6 genotype to phenotype translation: Consensus recommendations from the Clinical Pharmacogenetics Implementation Consortium and Dutch Pharmacogenetics Working Group. Clin Transl Sci. 2020;13:116-124. doi: 10.1111/cts.12692.

2. Zeng W, Liu H, Malla P, Zhao Y, Menezes L, Cao Y, Wang C, Yang Z, Cui L. Efficacy of primaquine for the radical cure of *Plasmodium vivax* malaria in northeast Myanmar and the impact of cytochrome P450 2D6 genotypes. Clin Infect Dis. 2025:ciae482. doi: 10.1093/cid/ciae482.

3. Sutanto I, Soebandrio A, Ekawati LL, Chand K, Noviyanti R, Satyagraha AW, Subekti D, Santy YW, Crenna-Darusallam C, Instiaty I, Budiman W, Prasetya CB, Lardo S, Elyazar I, Duparc S, Cedar E, Rolfe K, Fernando D, Berni A, Jones S, Kleim JP, Fletcher K, Sharma H, Martin A, Taylor M, Goyal N, Green JA, Tan LK, Baird JK. Tafenoquine co-administered with dihydroartemisinin-piperaquine for the radical cure of *Plasmodium vivax* malaria (INSPECTOR): a randomised, placebo-controlled, efficacy and safety study. Lancet Infect Dis. 2023;23:1153-1163. doi: 10.1016/S1473-3099(23)00213-X.

4. Baird JK, Louisa M, Noviyanti R, Ekawati L, Elyazar I, Subekti D, Chand K, Gayatri A, Instiaty, Soebianto S, Crenna-Darusallam C, Djoko D, Hasto BD, Meriyenes D, Wesche D, Nelwan EJ, Sutanto I, Sudoyo H, Setiabudy R. Association of impaired cytochrome P450 2D6 activity genotype and phenotype with therapeutic efficacy of primaquine treatment for latent *Plasmodium vivax* malaria. JAMA Netw Open. 2018;1:e181449. doi: 10.1001/jamanetworkopen.2018.1449.

5. Brasil LW, Rodrigues-Soares F, Santoro AB, Almeida ACG, Kühn A, Ramasawmy R, Lacerda MVG, Monteiro WM, Suarez-Kurtz G. CYP2D6 activity and the risk of recurrence of *Plasmodium vivax* malaria in the Brazilian Amazon: a prospective cohort study. Malar J. 2018;17:57. doi: 10.1186/s12936-017-2139-7.

6. Silvino ACR, Kano FS, Costa MA, Fontes CJF, Soares IS, de Brito CFA, Carvalho LH, Sousa TN. Novel insights into *Plasmodium vivax* therapeutic failure: CYP2D6 activity and time of exposure to malaria modulate the risk of recurrence. Antimicrob Agents Chemother. 2020;64:e02056-19. doi: 10.1128/AAC.02056-19.

7. da Silva GS, Fontenelle FA, Carvalho AO, Macêdo MM, Morais MC, Abreu Netto RL, Mwangi VI, Alecrim MGC, Lacerda MVG, Rodrigues-Soares F, de Almeida ACG, de Melo GC. Impact of CYP2D6, MAOA, and UGT2B7 genetic variants on recurrence of *Plasmodium vivax* in the Brazilian Amazon. Sci Rep. 2025;15:15330. doi: 10.1038/s41598-025-94679-7.

8. Sierra-Cifuentes V, Zuluaga-Idárraga L, Aguirre-Acevedo D, Silva de Barros Puça MC, Nobrega de Sousa T, Lopera-Mesa TM. Polymorphisms of the CYP2D6 gene and its relationship with *Plasmodium vivax* relapses after chloroquine-primaquine treatment in Turbo, Colombia. Diagn Microbiol Infect Dis. 2025;113:117013. doi: 10.1016/j.diagmicrobio.2025.117013.

9. Chamma-Siqueira NN, Negreiros SC, Ballard SB, Farias S, Silva SP, Chenet SM, Santos EJM, Pereira de Sena LW, Póvoa da Costa F, Cardoso-Mello AGN, Marchesini PB, Peterka CRL, Viana GMR, Macedo de Oliveira A. Higher-dose primaquine to prevent relapse of *Plasmodium vivax* malaria. N Engl J Med. 2022;386:1244-1253. doi: 10.1056/NEJMoa2104226.

10. Stern S, Hyland PL, Pacanowski M, Schuck RN. Leveraging in vitro models for clinically relevant rare CYP2D6 variants in pharmacogenomics. Drug Metab Dispos. 2024;52:159-170. doi: 10.1124/dmd.123.001512.

11. Zhou Y, Lauschke VM. Population pharmacogenomics: an update on ethnogeographic differences and opportunities for precision public health. Hum Genet. 2022;141:1113-1136. doi: 10.1007/s00439-021-02385-x.

12. Choi S, Choi H, Park SY, Kwak YG, Song JE, Shin SY, Baek JH, Shin HI, Cho SH, Lee SE, Kwon JR, Park S, Kim M, Oh HS, Kim YC, Kim MJ, Yeom JS. Association between CYP2D6 phenotype and recurrence of *Plasmodium vivax* infection in south Korean patients. Malar J. 2022;21:289. doi: 10.1186/s12936-022-04311-6.

13. Llanos-Cuentas A, Manrrique P, Rosas-Aguirre A, Herrera S, Hsiang MS. Tafenoquine for the treatment of *Plasmodium vivax* malaria. Expert Opin Pharmacother. 2022;23:759-768. doi: 10.1080/14656566.2022.2058394.

14. Suarez-Kurtz G. Impact of CYP2D6 genetic variation on radical cure of *Plasmodium vivax* malaria. Clin Pharmacol Ther. 2021;110:595-598. doi: 10.1002/cpt.2313.

15. Marcsisin SR, Sousa JC, Reichard GA, Caridha D, Zeng Q, Roncal N, McNulty R, Careagabarja J, Sciotti RJ, Bennett JW, Zottig VE, Deye G, Li Q, Read L, Hickman M, Dhammika Nanayakkara NP, Walker LA, Smith B, Melendez V, Pybus BS. Tafenoquine and NPC-1161B require CYP 2D metabolism for anti-malarial activity: implications for the 8-aminoquinoline class of anti-malarial compounds. Malar J. 2014;13:2. doi: 10.1186/1475-2875-13-2.

16. Milner EE, Berman J, Caridha D, Dickson SP, Hickman M, Lee PJ, Marcsisin SR, Read LT, Roncal N, Vesely BA, Xie LH, Zhang J, Zhang P, Li Q. Cytochrome P450 2D-mediated metabolism is not necessary for tafenoquine and primaquine to eradicate the erythrocytic stages of *Plasmodium berghei*. Malar J. 2016;15:588. doi: 10.1186/s12936-016-1632-8.

17. St Jean PL, Xue Z, Carter N, Koh GC, Duparc S, Taylor M, Beaumont C, Llanos-Cuentas A, Rueangweerayut R, Krudsood S, Green JA, Rubio JP. Tafenoquine treatment of *Plasmodium vivax* malaria: suggestive evidence that CYP2D6 reduced metabolism is not associated with relapse in the Phase 2b DETECTIVE trial. Malar J. 2016;15:97. doi: 10.1186/s12936-016-1145-5.

18. Ministry of Health of Brazil. Practical guidelines for malaria therapy [in Portuguese]. Brasília, Ministry of Health of Brazil, 2010. Available from: http://bvsms.saude.gov.br/bvs/publicacoes/guia_pratico_malaria.pdf

19. Negreiros S, Farias S, Viana GM, Okoth SA, Chenet SM, de Souza TM, Marchesini P, Udhayakumar V, Povoa MM, Santelli AC, de Oliveira AM. Efficacy of chloroquine and primaquine for the treatment of uncomplicated *Plasmodium vivax* malaria in Cruzeiro do Sul, Brazil. Am J Trop Med Hyg. 2016;95:1061-1068. doi: 10.4269/ajtmh.16-0075.

20. Ladeia-Andrade S, Menezes MJ, de Sousa TN, Silvino ACR, de Carvalho JF Jr, Salla LC, Nery OA, de Melo GNP, Corder RM, Rodrigues PT, Ferreira MU. Monitoring the efficacy of chloroquine-primaquine therapy for uncomplicated *Plasmodium vivax* malaria in the main transmission hot spot of Brazil. Antimicrob Agents Chemother. 2019;63:e01965-18. doi: 10.1128/AAC.01965-18.

21. Itoh M, Negreiros do Valle S, Farias S, Holanda de Souza TM, Rachid Viana GM, Lucchi N, Chenet S, Marchesini P, Póvoa M, Faria e Silva Santelli AC, Macedo de Oliveira A. Efficacy of artemether-lumefantrine for uncomplicated *Plasmodium falciparum* malaria in Cruzeiro do Sul, Brazil, 2016. Am J Trop Med Hyg. 2018;98:88-94. doi: 10.4269/ajtmh.17-0623.15.

22. Ladeia-Andrade S, de Melo GN, de Souza-Lima RC, Salla LC, Bastos MS, Rodrigues PT, Luz FC, Ferreira MU. No clinical or molecular evidence of *Plasmodium falciparum* resistance to artesunate-mefloquine in northwestern Brazil. Am J Trop Med Hyg. 2016;95:148-54. doi: 10.4269/ajtmh.16-0017.

23. Puça MCSB, Naziazeno IM, Fernandes Dos Santos VC, Rodrigues P, Calil PR, Ladeia WA, Gil JP, Ferreira MU, Nobrega de Sousa T. How CYP2D6 polymorphism modulates the community-wide risk of *Plasmodium vivax* infection: a panel study in Amazonian Brazil. J Infect Dis. 2025:jiaf412. doi: 10.1093/infdis/jiaf412.

24. Stephens M, Smith NJ, Donnelly P. A new statistical method for haplotype reconstruction from population data. Am J Hum Genet. 2001;68:978-89. doi: 10.1086/319501

25. King CL, Adams JH, Xianli J, Grimberg BT, McHenry AM, Greenberg LJ, Siddiqui A, Howes RE, da Silva-Nunes M, Ferreira MU, Zimmerman PA. Fy(a)/Fy(b) antigen polymorphism in human erythrocyte Duffy antigen affects susceptibility to *Plasmodium vivax* malaria. Proc Natl Acad Sci U S A. 2011;108:20113-8. doi: 10.1073/pnas.1109621108.

26. Kempińska-Podhorodecka A, Knap O, Drozd A, Kaczmarczyk M, Parafiniuk M, Parczewski M, Ciechanowicz A. Analysis for genotyping Duffy blood group in inhabitants of Sudan, the fourth cataract of the Nile. Malar J. 2012;11:115. doi: 10.1186/1475-2875-11-115.

27. Baroni L, Pedroso M, Barcellos C, Salles R, Salles S, Paixão B, Chrispino A, Guedes G, Ogasawara E. An integrated dataset of malaria notifications in the Legal Amazon. BMC Res Notes. 2020;13:274. doi: 10.1186/s13104-020-05109-y.

28. Daher A, Silva JCAL, Stevens A, Marchesini P, Fontes CJ, ter Kuile FO, Lalloo DG. Evaluation of *Plasmodium vivax* malaria recurrence in Brazil. Malar J. 2019;18:18. doi: 10.1186/s12936-019-2644-y.

29. Vera-Arias CA, Holzschuh A, Oduma CO, Badu K, Abdul-Hakim M, Yukich J, Hetzel MW, Fakih BS, Ali A, Ferreira MU, Ladeia-Andrade S, Sáenz FE, Afrane Y, Zemene E, Yewhalaw D, Kazura JW, Yan G, Koepfli C. High-throughput *Plasmodium falciparum* *hrp2* and *hrp3* gene deletion typing by digital PCR to monitor malaria rapid diagnostic test efficacy. Elife. 2022;11:e72083. doi: 10.7554/eLife.72083.

30 Prete CA Jr, de Sousa TN, Naziazeno IM, Puça MCSB, Ladeia WA, Rodrigues PT, Johansen IC, Paula GA, Ferreira MU, Corder RM; Mâncio Lima Cohort Study Working Group. How Duffy blood group (FY) polymorphism and age modulate vivax malaria risk at the community level: a population-based retrospective cohort study in the Amazon. J Infect Dis. 2025:jiaf562. doi: 10.1093/infdis/jiaf562

31. Winkler W. String comparator metrics and enhanced decision rules in the Fellegi-Sunter model of record linkage. American Statistical Association 1990 Proceedings of the Section on Survey Research Methods, pp. 354–359.

32. Corder RM, de Lima ACP, Khoury DS, Docken SS, Davenport MP, Ferreira MU. Quantifying and preventing *Plasmodium vivax* recurrences in primaquine-untreated pregnant women: An observational and modeling study in Brazil. PLoS Negl Trop Dis. 2020; 14:e0008526. doi: 10.1371/journal.pntd.0008526

33. Marcelino D. SoundexBR: Soundex (Phonetic) Algorithm for Brazilian Portuguese. R package version 1.2, <http://CRAN.R-project.org/package=SoundexBR>, 2015.

34. Boulos M, Amato Neto V, Dutra AP, di Santi SM, Shiroma M. Frequency of malaria relapse due to *Plasmodium vivax* in a non-endemic region (São Paulo, Brazil) [in Portuguese]. Rev Inst Med Trop Sao Paulo. 1991;33:143-6.

35. Garcia KKS, de Deus Henriques KM, da Silva Balieiro AA, de Pina-Costa A, Siqueira AM. Towards malaria elimination: a case-control study to assess associated factors to malaria relapses in the extra-Amazon Region of Brazil from 2008 to 2019. Malar J. 2024;23:312. doi: 10.1186/s12936-024-05133-4.

36. Pedro RS, Guaraldo L, Campos DP, Costa AP, Daniel-Ribeiro CT, Brasil P. *Plasmodium vivax* malaria relapses at a travel medicine centre in Rio de Janeiro, a non-endemic area in Brazil. Malar J. 2012;11:245. doi: 10.1186/1475-2875-11-245.

37. Battle KE, Karhunen MS, Bhatt S, Gething PW, Howes RE, Golding N, Van Boeckel TP, Messina JP, Shanks GD, Smith DL, Baird JK, Hay SI. Geographical variation in *Plasmodium vivax* relapse. Malar J. 2014;13:144. doi: 10.1186/1475-2875-13-144.

38. Corder RM, Paula GA, Pincelli A, Ferreira MU. Statistical modeling of surveillance data to identify correlates of urban malaria risk: A population-based study in the Amazon Basin. PLoS One. 2019;14:e0220980. doi: 10.1371/journal.pone.0220980.

39. Douglas NM, Lampah DA, Kenangalem E, Simpson JA, Poespoprodjo JR, Sugiarto P, Anstey NM, Price RN. Major burden of severe anemia from non-falciparum malaria species in Southern Papua: a hospital-based surveillance study. PLoS Med. 2013;10:e1001575.

40. Pincelli A, Cardoso MA, Malta MB, Johansen IC, Corder RM, Nicolete VC, Soares IS, Castro MC, Ferreira MU; MINA-Brazil Study Working Group. Low-level *Plasmodium vivax* exposure, maternal antibodies, and anemia in early childhood: Population-based birth cohort study in Amazonian Brazil. PLoS Negl Trop Dis. 2021;15:e0009568. doi: 10.1371/journal.pntd.0009568.

41. Van den Eede P, Soto-Calle VE, Delgado C, Gamboa D, Grande T, Rodriguez H, Llanos-Cuentas A, Anné J, D'Alessandro U, Erhart A. *Plasmodium vivax* sub-patent infections after radical treatment are common in Peruvian patients: results of a 1–year prospective cohort study. PLoS One. 2011;6:e16257.
