# Supplementary material for: Reduced cytochrome P-450 (CYP) 2D6 activity and Plasmodium vivax malaria risk in Amazonians: A retrospective, population-based cohort study
Source: PLoS Negl Trop Dis. 2026 Mar 27;20(3):e0014160. doi: 10.1371/journal.pntd.0014160 (PMC13048497; doi:10.1371/journal.pntd.0014160)
Supplement: Attachment — Submitted filename: Rebuttal letter.pdf [file pntd.0014160.s019.pdf]

Re: PNTD-D-25-01961
Reduced cytochrome P-450 (CYP) 2D6 activity and Plasmodium vivax malaria risk in Amazonians: a retrospective, population-based cohort study

We sincerely appreciate the very careful review, with numerous constructive comments and suggestions by the Academic Editor of *PLoS Neglected Infectious Diseases* and the reviewer, here highlighted in blue. We have addressed all concerns raised by them and extensively revised the manuscript.

Academic Editor:

1. In the reference list, provide the names of up to 6 authors (followed by "et al." if there are more than 6 authors). Use en dashes for page number ranges, not hyphens. As per the journal's style, "abbreviate" the second page number in those references where it does not need to appear in full (as has been done in line 463). E.g., in line 382, write "116–24", as opposed to "116-124"; and in line 387, "595–8" instead of "595-598". At the end of references where the DOI is given, omit the full stop (period) behind the DOI, as at the end of line 441.

Corrected, thanks!

2. "time-to-recurrence" is correctly used adjectivally (meaning that there are hyphens) in lines 131, 258 and 309. But there is lack of uniformity, hyphen-wise, where the expression appears non-adjectivally in lines 195 and 254. There are no hyphens in line 195 (which is in order, because that is correct) but they are present in line 254. Delete the hyphens in line 254.

Done.

3. "CYP2D6" sometimes appears in italics and sometimes it doesn't (mostly). Uniformity is needed. I suggest no italics, partly to avoid having to decide about "P450 (CYP)". But in the reference list, follow what was in the original publications.

In the main text, we kept the italics to refer to the *CYP2D6* gene (e.g., *CYP2D6* genotype, *CYP2D6* alleles etc.) instead of to the CYP2D6 gene product (enzyme; e.g., CYP2D6 activity), as usually recommended in scientific literature. We carefully revised the whole manuscript to ensure a consistent use of italics. As suggested, we kept the italics in the references according to authors' use.

4. Both "Figure" and "Fig." appear in the manuscript (MS) (and in line 177, "Fig" does not have a full stop/period after it, unlike elsewhere). Introduce uniformity by checking the MS in this respect. Copy the style used in some recent publications in the journal.

Corrected.
5. Line (L) 3 to L 4: *Inter alia*, primaquine *per se* not being a "treatment", reword the text something like: "Primaquine (PQ) is the only widely available drug that eliminates latent hypnozoites, thereby preventing relapses …".

Corrected.

6. L 4: In view of the fact that it is technically the infection which relapses, not the parasite (*Plasmodium vivax*), substitute "of *Plasmodium vivax* malaria" for "of the human malaria parasite *Plasmodium vivax*".

Corrected.

7. L 5: Insert a hyphen between "P" and "450", as has been done elsewhere, for uniformity.

Done.

8. L 6: Correct "minimal" to "minimum" (there being a subtle difference in meaning in regard to this sentence).

Done.

9. L 33: Substitute "drug that eliminates" for "treatment to eliminate"; and insert a comma at the end of the line, after "hypnozoites".

Done.

10. L 34: Substitute "thus preventing" for "and prevent".

Done.

11. L 34: Substitute "of *Plasmodium vivax* malaria" for "of the human malaria parasite *Plasmodium vivax*". The reason is that, as stated in one of the comments above, *Plasmodium vivax per se* (i.e., the parasite itself) does not relapse.

Done.

12. L 36: Correct "minimal" to "minimum". The reason is given in comment no. 8, above.

Corrected.

13. L 46: The word "neglected" used to be appropriate for *P. vivax* but in 2026 is doubtfully applicable, depending on how one views the meaning of "neglected". A lot of research involving *P. vivax* is being carried out these days. Therefore, consider deleting "neglected". But leave it there if you like it.

We changed to "relatively neglected" to highlight that *P. vivax* remains relatively neglected, compared to *P. falciparum*.

14. L 47 & L 114, Etc.: Abbreviate "*Plasmodium vivax*" as "*P. vivax*" in these two lines. But in line 323 (heading for "S5 Table") as well as in the headings for Tables 3 and 4, "*Plasmodium*" should appear in full, as it does in other headings.

Corrected.

15. L 49: Substitute "give rise to" for "originates". Also in this line, delete "repeated", because coming after "one or more", it is saying the same thing twice.

Done

16. L 49: With reference to what one of the reviewers wrote, insert “symptomatic” in front of “malaria” here (and elsewhere in the MS, where appropriate).

In our study, nearly all malarial infections recorded were symptomatic, because diagnosis is routinely made through passive case detection. However, when we refer to relapses in general, as in L 49, most of them are asymptomatic (e.g., DOI: 10.1371/journal.pone.0016257). Therefore, we kept the “malaria” here (without “asymptomatic”), but changed other parts of the text (e.g., “symptomatic recurrence” at the end of the introduction) when we referred to the relapses detected by passive case detection. We chose to use “clinical malaria” most of the times. We added a brief explanation to the study outcome definition.

17. L 49: Delete “repeated” because coming after “one or more”, it is saying the same thing twice.

Done.

18. L 50: It is suggested that the comma behind “episodes” be deleted and that “often occurring” be inserted in front of “several”, because relapses can also take place outside the range given in line 50.

Done.

19. L 56: Add an s” to “account” to make it “accounts” (the antecedent in the sentence being “activity”).

Actually, “account” refers to “metabolites”, not to “redox activity”, since the metabolites (and not necessarily the fact that they have redox activity) account for PQ activity. We thus kept “account”.

20. L 56: After “[5]” in this line, consider inserting a sentence such as: “It has recently been suggested that, because of this mechanism of action [5], the massive, newly discovered, hidden extrahepatic reservoir of asexual *P. vivax* parasites in the spleen and bone marrow might (also) be targeted by primaquine [REF.]”. The sole original primaquine-associated reference here is PMID 31522991 (alternatively or additionally, PMID 10223033). The relevance hereof is that this mode of parasite inactivation, if it does take place to a significant extent (synergistically or otherwise with especially chloroquine; and for the logical biochemical reasons concerned), will obfuscate what the number of relapses is, because recrudescences will also be prevented. But by including a sentence like this, the authors would then be covered in regard to what the MS otherwise states overall, considering that the asexual parasite inhibition matter will have been mentioned in passing (as above). [You referred previously to this splenic parasite accumulation phenomenon in the paper you have self-cited in this MS as reference 16.]

We added the reference as suggested.

21. L 68 & L 69: Alter “[9-11]” in this line to “[9–11]” and “[12-14]” in the next line to “[12–14]”. In other words, substitute en dashes for the hyphens. An en dash is used for a reference range, not a hyphen.

Corrected.

22. L 97: Check whether you do wish to use italics for "*ACKR1*".

In this case, *ACKR1* refers to the gene, not to the gene product, hence the italic is appropriate.

23. L 101: "whatever" should be changed to "whichever" because you are referring back to specific options here.

Corrected.

24. L 112: "malaria infection" appears in this line. Although likewise to be found in publications frequently, it is nevertheless a misnomer. "Malaria" is by definition an infection, so to add "infection" after it is amounts to saying the same thing twice. Thus, "infection" behind "malaria" is superfluous. Either delete "malaria" in this line or leave it there and substitute "case" for "infection" (assuming that this is what you mean).

Corrected.

25. L 112: Consider inserting "plasmodial" between "different" and "species".
Done.

26. L 113: Insert "had" after "have" so that the wording becomes "have had".

Done.

27. L 114: Abbreviate "*Plasmodium*" as "*P.*", as requested in comment no. 14.

Done.

28. L 115: Consider inserting "those of" after "such as".

Done.

29. L 131: Correct "run" to "ran".

Done.

30. L 132: Insert "episode" between "malaria" and "experienced".

Done.

31. L 133: Insert "drug" between "partner" and "other".

Done.

32. L 134: Insert "malarial" in front of "recurrence" because "*P. vivax* recurrence" is incorrect (it is not the parasite that recurs, as already pointed out in other comments here).

We added "malaria".

33. L 137 & L 139: Should “site” in these two lines be “area”? Where the study was carried out seems larger than a “site”.

Corrected to “area”.

34. L 137: Consider altering the “is unlikely” assertion to the less definite “seems unlikely”.

Done.

35. L 138: Substitute “did not suffer” (or “did not experience”) for “remained free of”, which is not a good expression to use here.

Done.

36. L139: Change the en dash in “180–day” (which is not a range, for which an en dash would have been correct) to a hyphen (“180-day”).

Done.

37. L 139: Insert “period” behind “follow-up”. The way it currently reads in what one would say in conversation (insufficiently formal for a publication).

Done.

38. L 140: Change “whatever” to “whichever”.

Done.

39. L 156: Alter “differed in” to “differed as regards”.

Done.

40. L. 156: Insert “an” in front of “individual’s”.

Done.

41. L 185: Correct “rising” to “raising”.

Done.

42. L 190: Substitute “recurrence-free” for “free of recurrence”.

Done.

43. L 198: Substitute “following infection with *P. vivax*” for “following a *P. vivax* infection”.

Done.

44. L 205: Correct the en dash in “person–years” to a hyphen (“person-years”).

Done.

45. L 224: Delete “a” in front of “severity” (it is subtly incorrect English to use “a” there).

Done.

46. L 228: Insert “of” between “20%” and “high-risk”.

Done.

47. L 234: Delete “a” in front of “normal”, to correct the English (“a” does not go together with “activity” in this sentence).

Done.

48. L 249: At the beginning of the line, delete “A” in front of “reduced”, to correct the English.

Done.

49. L 249: Correct “minimal” at the end of the line to “minimum”.

Done.

50. L 251: Correct the sentence by inserting “malaria” after “*P. vivax*”.

Done.

51. L 259: Substitute “that causes” for “causing” (finite verb needed instead of present participle).

Done.

52. L 263: Delete “a” in front of “CYP2D6”.

Done.

53. L 272 & L 273: You could leave “(less likely)” there if that is what you believe because nobody is about to prove you wrong. However, other possibilities are to, more safely, insert “perhaps” in front of “less”; or, even more safely, to delete “(less likely)”.

Given the high CQ-PQ efficacy for clearing blood-stage *P. vivax* parasitemia in this area, we believe that “less likely” remains appropriate.

54. L 273: It has become apparent that it is highly likely, parasitologically, that both early and late *P. vivax* recrudescences take place. To cover the authors and also to reflect their awareness, consider inserting

PMID 28366603 (key original reference for this probability) after "late recrudescences" in this line. Thus, "… late recrudescences [REF.] collectively defined …".

55. L 273: Think about whether your paper would be academically enhanced by adding the following recent (2026) citation in this line at the end of the sentence after "recurrences": PMID 41324557. The publication fits in with your statement about not separating recurrences into 3 categories, by showing how it can be attempted under the right circumstances. Citation of the article would at the same time provide the reader with a topical reference that gives further insight into your definition of "recurrences". Thus, "… collectively defined as recurrences [REF.]. Relapse frequencies were not …".

We added two references [32,33] that describe modelling approaches to distinguish between relapses and new infections.

56. L 274: Delete "the" in front of "adherence". This is an example in English of where omission of "the" is grammatically and logically correct (unlike instances where "the" should indeed appear).

Done.

57. L 278: Substitute a full stop for the comma after "1.0"; delete "with" in the line; and start a new sentence by inserting "This has" in front of "practical".

Done.

58. L 283: It looks as though there is a space which should not be there between "Calil" and the comma that follows.

Corrected.

59. L 308: Insert "drug" between "partner" and "other".

Done.

60. L. 309: Substitute "131 first or only *P. vivax* malaria recurrences" for "131 *P. vivax* first or only recurrences".

Done.

61. L 310: Insert "period" after "follow-up". The latter on its own is too informal for a publication such as this one.

Done.

62. L 321: Substitute "2014–2018" for "2014-18", using an en dash, not a hyphen, because it is a range (for which en dashes are used rather than hyphens).

Done.

63. L 323: Insert “malaria” after “*P. vivax*” (before “recurrence”).

Done.

64. L 324. Alter “2014-18” as described in the comment for line 321.

Done.

65. L 325: Is something missing behind “**S1 File**”?

We added “Supplementary Methods, Results, and Discussion”.

66. L 354: Although “Secretaria” might, I dare say, be used for the department or office (“team” in line 353), translation into English would make sense to me as “at the Health Secretariat” (the equivalent in English), rather than “at the Health Secretary” (considering that in English, “Secretary” is a single person). I suggest that you alter “Secretary” to “Secretariat”.

Corrected.

67. L 368 to L 370: Citation of the 2024 World Malaria Report is fine. But should the same information in respect of why you are citing it happen to be in the 2025 report, that would potentially be citable (as a more recent report): https://www.who.int/publications/i/item/9789240117822

The 2024 report was the latest available at the time the manuscript was written. We changed the reference to the mos recent (2025) report.

68. L 381: For uniformity, use a lower case “c” in “consensus (after the colon), as has been done after colons elsewhere in the reference list.

Done.

69. L 385: Alter the hyphen in “1381-2” to an en dash (“1381–2”) because it is a page range in this instance. Actual page numbers are involved, not an article number (as in line 391, for example, where the hyphen is correct). The abbreviated ”1381–2” is correct in this line, being the journal’s style (not the “1381–1382” style used for various other references listed and in respect of which changes by the authors have been requested via the first comment in this editor’s report).

Done.

70. L 392: Insert a hyphen between the “S” and “B” after “Ballard” to change from “SB” to “S-B”.

Done.

71. L 394: Use an upper case first letter in “higher” so that it becomes “Higher”. [The “A” in front of it is not the beginning of a sentence, but an initial of an author.]

Done.

72. L 416: Substitute “Rodrigues PT” for “Rodrigues P” (add “T” as a second initial, which is in the original publication).

Done.

73. L 417: Just as you used the italics that were in the title of the original reference 14 paper, put “CYP2D6” in this line in italics.

Done.

74. L 418: Substitute “2025;232:e571–9” for “2025 Aug 19:jiaf412”.

Done.

75. L 425: Use an upper case “U” in “urban” (as in the original publication).

Done.

76. L 433: Extend the initials for author Viana to “GMR”.

Done.

77. L 453: Substitute “2025;81:379–86” for “2025:ciae482”.

Done.

78. Headings for Table 1, 2, 3, 4 & 5 in main MS: In each table, change “2014-18” to “2014–2018, using an en dash instead of a hyphen.

Done.

79. In the heading for Table 2 in the main MS, insert “malaria” between “*P. vivax*” and “recurrence”.

Done.

80. Reminder: Reduce the number of authors’ names given in the reference list in accordance with comment no. 1 here.

Done.

Reviewer #1:

This is a very clearly written and concise manuscript showing that in Mâncio Lima, Brazil, among 466 people with at least one mono-species *P. vivax* infection detected in Brazil’s SIVEP-Malaria database, people with a null / low CYP2D6 activity score (AS) have a higher risk of recurrence. However, among all 997 study participants, the incidence of *P. vivax* cases detected in Brazil’s SIVEP-Malaria database is not higher among people with null / low AS.

Thanks for the positive comments!

The authors report that 98.5% of cases in the SIVEP-Malaria database are symptomatic. As such, all results essentially condition on infection being symptomatic. Having a symptomatic infection is an indicator of not having complete immunity. Among the 466 study participants with this indicator of non-immunity, people with null / low CYP2D6 activity have a higher risk of recurrence. This makes sense. So too does the non-significant interaction between age and AS: among people who have already been selected for using an indicator of non-immunity there is little immune variance left for age to be a proxy of. Incidence is computed from all 997 study participants, including people with null / low AS and zero SIVEP-Malaria infection. These people might have elevated immunity due to null / low AS exposing them to more recurrence in the past.

Major comments

1. Among the 70 people with null / low AS, does the distribution of ages among the 34 with at least one SIVEP-Malaria database infection differ to the distribution among the 36 without a SIVEP-Malaria infection?

Actually, there is no difference in mean ages: t = -0.087622, df = 67.192, p-value = 0.9304

Age (in years) of people with no recurrence:
1.008219 1.835616 2.947945 3.054795 3.997260 4.660274 5.800000 7.723288 9.260274 11.547945 12.126027 12.397260 12.695890 13.043836 16.901370 17.394521 17.967123 19.997260 22.484932 30.882192 30.967123 31.654795 31.775342 31.802740 33.405479 33.608219 37.054795 39.408219 42.252055 43.490411 44.783562 52.701370 52.756164 71.578082 82.972603 84.832877

Age (in years) of people with recurrence:
2.553425 5.594521 7.997260 8.194521 8.597260 10.468493 10.997260 11.652055 12.887671 14.931507 15.443836 15.772603 15.830137 15.997260 15.997260 16.386301 18.008219 23.813699 25.852055 29.723288 32.060274 32.134247 33.279452 35.602740 37.465753 38.583562 41.838356 42.230137 49.271233 54.726027 55.805479 56.912329 58.709589 77.947945

2. Following from (1) did the authors consider a lower upper bound for the youngest age group? This seems reasonable given that the prevalence result reported on line 233-235 (which includes asymptomatic) suggests the impact of null / low AS on bolstering immunity is already detectable by adolescence. If evidence for a different age distribution is seen among the 36 vs 34 people, the upper bound of the lowest age category could be set lower than some low summary statistic of the distribution among the 36 people with zero SIVEP-Malaria infection.

We used the same age groups as in another study on the same cohort population (doi: 10.1093/infdis/jiaf562) that shows a significant interaction between age and Duffy genotype. Unsurprisingly, the 6-15-years group has a higher vivax malaria incidence than the 0-5-years group (e.g., doi: 10.1371/journal.pone.0220980, Figure 1B)

3. Since the authors essentially analyse only symptomatic infection, I suggest adding symptomatic throughout (see below). More stringently, the authors could consider removing the 1.5% non-symptomatic from the analyses, without large loss of data.

Yes, this is correct! We added the following clarification to our definition of study outcome (Methods, Retrospective Cohort, second paragraph): "The study outcome was any single-species *P. vivax* infection, irrespective of parasite density and the presence of symptoms, diagnosed by thick-smear microscopy or (rarely) by rapid diagnostic test from January 1, 2014, through December 31, 2018. Because the vast majority of infections were symptomatic (see Results), we can alternatively define our study outcome as vivax malaria – i.e., symptomatic infection with *P. vivax*.".

4. Given age as a proxy of immunity is expected to decrease malaria risk, but age as a proxy for occupational exposure is expected to increase risk, it would be nice to discuss the potential benefit of serological data.

We acknowledge that failing to examine correlates of clinical immunity (e.g., specific antibody levels) is a study limitation. We added the following sentence to the last but one paragraph (study limitations): "Fourth, we assume that cohort participants gradually developed clinically immunity as they become more exposed to malaria with increasing age, but did not measure laboratory correlates of acquired immunity (e.g., levels of IgG antibodies to key *P. vivax* antigens) to test this hypothesis in the study population."

Minor suggestions

5. Line 165: is there a way to indicate how the 643 Fy-positive people are distributed across the excluded participants in Fig S2? Also, please state in the main text why the 643 people were not included

   Thanks for pointing this out. We realized that S2 Fig, should be corrected, because it did not reflect the order of exclusions. We revised S2 Fig. in order to show that we first excluded 680 individuals who had been genotyped for Duffy but not for CYP2D6, next we excluded 106 study participants that had been genotyped for CYP2D2 but were Duffy-negative. The revised S2 Fig. shows these steps. We added to the main text a brief description of these exclusions.

6. Line 197: "may suffice for PQ activation and anti – symptomatic - relapse efficacy in the context of this study" (higher hypnozoite burdens elsewhere might require higher AS)

   Added "symptomatic" as suggested.

7. Line 198: "significantly increased risk of symptomatic recurrence"

   Added "symptomatic" as suggested.

8. Line 203: "incidence rates of symptomatic P. vivax infection"

   Added "symptomatic" as suggested.

9. Line 215-216: "The association between […] and age on symptomatic vivax malaria"

   We interpret that malaria refers to the disease (i.e., symptomatic infection), not to any plasmodial infection regardless of any clinical manifestations (e.g., "malaria: a serious disease that causes a high temperature and shivering (= shaking of the body) caused by the bite of some

types of mosquito"— Oxford Learner's Dictionary, https://www.oxfordlearnersdictionaries.com/us/definition/english/malaria#google_vignette). For this reason, we kept "malaria" here, instead of "symptomatic malaria". We had clarified this use of "malaria" as a symptomatic infection in the Methods section (see comment #3).

10. Line 224: "<u>symptomatic</u> vivax malaria recurrence"

    Please see the comment above.

11. Line 249-250: since different settings with different liver burdens likely have different minima, consider making it plural, e.g., "but the minimal level<u>s</u> […] needed <u>across different settings</u>…"

    Thanks for the suggestion. Done!

12. Line 259: likewise, "level<u>s</u>"

    Done.

13. S1 File, Section 14: "However, once infected <u>symptomatically</u>"

    Done.

14. Table 2: The numbers in second column are incorrect: they sum to 997 not 446

    We changed the format of Table 2 in order to make clear that rows, not columns, should be summed to obtain the total (n = 446). Note that numbers in columns are cumulative (e.g., the 70 participants below the 0.25 AS threshold include 45 participants with zero activity and so on).

S1 File:

15. Literature review: across misspelt acroos

Corrected!

16. Marcsisin et al ref 15 missing right square bracket

Corrected!

17. Section 7: phenotypes inferred from genotypes

Corrected!

18. Section 14, first line: add 997 to "all <u>997</u> study participants,"

Done.

19. Section 14, second paragraph: Table 4 not 3, main text.

Corrected.

Journal Requirements:

1) Please ensure that the CRediT author contributions listed for every co-author are completed accurately and in full.

At this stage, the following Authors/Authors require contributions: Marcelo U. Ferreira. Please ensure that the full contributions of each author are acknowledged in the "Add/Edit/Remove Authors" section of our submission form. The list of CRediT author contributions may be found here: https://journals.plos.org/plosntds/s/authorship#loc-author-contributions

We added the contribution of Marcelo U. Ferreira.

2) Tables should not be uploaded as individual files. Please remove these files and include the Tables in your manuscript file as editable, cell-based objects. For more information about how to format tables, see our guidelines: https://journals.plos.org/plosntds/s/tables

Done.

3) Some material included in your submission may be copyrighted. According to PLOSu2019s copyright policy, authors who use figures or other material (e.g., graphics, clipart, maps) from another author or copyright holder must demonstrate or obtain permission to publish this material under the Creative Commons Attribution 4.0 International (CC BY 4.0) License used by PLOS journals. Please closely review the details of PLOSu2019s copyright requirements here: PLOS Licenses and Copyright. If you need to request permissions from a copyright holder, you may use PLOS's Copyright Content Permission form.

Please respond directly to this email and provide any known details concerning your material's license terms and permissions required for reuse, even if you have not yet obtained copyright permissions or are unsure of your material's copyright compatibility. Once you have responded and addressed all other outstanding technical requirements, you may resubmit your manuscript within Editorial Manager.

Potential Copyright Issues:
i) Please confirm
(a) that you are the photographer of S1B, or (b) provide written permission from the photographer to publish the photo(s) under our CC BY 4.0 license.

Yes, the picture was taken by Rodrigo M. Corder, first author of the manuscript.

ii) Figure 1A. Please (a) provide a direct link to the base layer of the map (i.e., the country or region border shape) and ensure this is also included in the figure legend; and (b) provide a link to the terms of use / license information for the base layer image or shapefile. We cannot publish proprietary or copyrighted maps (e.g. Google Maps, Mapquest) and the terms of use for your map base layer must be compatible with our CC BY 4.0 license.

Figure created with QGIS software version 3.14, an open-source Geographic Information System (GIS) licensed under the GNU General Public License (https://bit.ly/2BSPB2F). Publicly available shape files provided from the Brazilian Institute of Geography and Statistics (IBGE) website (https://bit.ly/34gMq0S). All geographical data are used under the Creative Commons Attribution License (CC BY 4.0). We added this information to the figure legend.

4) Please note that your Data Availability Statement is currently missing the DOI/accession number of each dataset OR a direct link to access each dataset. If your manuscript is accepted for publication, you will be asked to provide these details on a very short timeline. We therefore suggest that you provide this information now, though we will not hold up the peer review process if you are unable.

We supply original data as S1 Database and S2 Database.

S1 Database. CSV file with all variables used in retrospective cohort study analysis.

S2 Database. CSV file with all variable used in the time-to-recurrence analysis.

5) In the online submission form, you indicated that Researchers who are interested in potential collaboration should contact the corresponding author (muferrei@usp.br).. All PLOS journals now require all data underlying the findings described in their manuscript to be freely available to other researchers, either

1. In a public repository
2. Within the manuscript itself
3. Uploaded as supplementary information.

This policy applies to all data except where public deposition would breach compliance with the protocol approved by your research ethics board. If your data cannot be made publicly available for ethical or legal reasons (e.g., public availability would compromise patient privacy), please explain your reasons by return email and your exemption request will be escalated to the editor for approval. Your exemption request will be handled independently and will not hold up the peer review process, but will need to be resolved should your manuscript be accepted for publication. One of the Editorial team will then be in touch if there are any issues.

See above.

6) Please amend your detailed Financial Disclosure statement. This is published with the article. It must therefore be completed in full sentences and contain the exact wording you wish to be published.
1) State the initials, alongside each funding source, of each author to receive each grant. For example: "This work was supported by the National Institutes of Health (####### to AM; ###### to CJ) and the National Science Foundation (###### to AM)."
2) State what role the funders took in the study. If the funders had no role in your study, please state: "The funders had no role in study design, data collection and analysis, decision to publish, or preparation of the manuscript."

3) If any authors received a salary from any of your funders, please state which authors and which funders..
If you did not receive any funding for this study, please simply state: The authors received no specific funding
for this work.
7) Please ensure that the funders and grant numbers match between the Financial Disclosure field and the Funding Inform

Done.
