# Supplementary material for: Reduced cytochrome P-450 (CYP) 2D6 activity and Plasmodium vivax malaria risk in Amazonians: A retrospective, population-based cohort study
Source: PLoS Negl Trop Dis. 2026 Mar 27;20(3):e0014160. doi: 10.1371/journal.pntd.0014160 (PMC13048497; doi:10.1371/journal.pntd.0014160)
Supplement: Attachment — Submitted filename: Review.docx [file pntd.0014160.s015.docx]

**Review**

This is a very clearly written and concise manuscript showing that in Mâncio Lima, Brazil, among 466 people with at least one mono-species *P. vivax* infection detected in Brazil’s SIVEP-Malaria database, people with a null / low CYP2D6 activity score (AS) have a higher risk of recurrence. However, among all 997 study participants, the incidence of *P. vivax* cases detected in Brazil’s SIVEP-Malaria database is not higher among people with null / low AS.

The authors report that 98.5% of cases in the SIVEP-Malaria database are symptomatic. As such, all results essentially condition on infection being symptomatic. Having a symptomatic infection is an indicator of not having complete immunity. Among the 466 study participants with this indicator of non-immunity, people with null / low CYP2D6 activity have a higher risk of recurrence. This makes sense. So too does the non-significant interaction between age and AS: among people who have already been selected for using an indicator of non-immunity there is little immune variance left for age to be a proxy of. Incidence is computed from all 997 study participants, including people with null / low AS and zero SIVEP-Malaria infection. These people might have elevated immunity due to null / low AS exposing them to more recurrence in the past.

**Major comments**

1. Among the 70 people with null / low AS, does the distribution of ages among the 34 with at least one SIVEP-Malaria database infection differ to the distribution among the 36 without a SIVEP-Malaria infection?
2. Following from (1) did the authors consider a lower upper bound for the youngest age group? This seems reasonable given that the prevalence result reported on line 233-235 (which includes asymptomatic) suggests the impact of null / low AS on bolstering immunity is already detectable by adolescence. If evidence for a different age distribution is seen among the 36 vs 34 people, the upper bound of the lowest age category could be set lower than some low summary statistic of the distribution among the 36 people with zero SIVEP-Malaria infection.
3. Since the authors essentially analyse only symptomatic infection, I suggest adding symptomatic throughout (see below). More stringently, the authors could consider removing the 1.5% non-symptomatic from the analyses, without large loss of data.
4. Given age as a proxy of immunity is expected to decrease malaria risk, but age as a proxy for occupational exposure is expected to increase risk, it would be nice to discuss the potential benefit of serological data.

**Minor suggestions**

5. Line 165: is there a way to indicate how the 643 Fy-positive people are distributed across the excluded participants in Fig S2? Also, please state in the main text why the 643 people were not included
6. Line 197: “may suffice for PQ activation and anti – symptomatic - relapse efficacy in the context of this study” (higher hypnozoite burdens elsewhere might require higher AS)
7. Line 198: “significantly increased risk of symptomatic recurrence”
8. Line 203: “incidence rates of symptomatic P. vivax infection”
9. Line 215-216: “The association between […] and age on symptomatic vivax malaria”
10. Line 224: “symptomatic vivax malaria recurrence”
11. Line 249-250: since different settings with different liver burdens likely have different minima, consider making it pleural, e.g., “but the minimal levels […] needed across different settings…”
12. Line 259: likewise, “levels”
13. S1 File, Section 14: “However, once infected symptomatically”
14. Table 2: The numbers in second column are incorrect: they sum to 997 not 446

S1 File:

15. Literature review: across misspelt acroos
16. Maracsisin et al ref 15 missing right square bracket
17. Section 7: phenotypes inferred from genotypes
18. Section 14, first line: add 997 to “all 997 study participants,”
19. Section 14, second paragraph: Table 4 not 3, main text.
