# Supplementary material for: Reduced cytochrome P-450 (CYP) 2D6 activity and Plasmodium vivax malaria risk in Amazonians: A retrospective, population-based cohort study
Source: PLoS Negl Trop Dis. 2026 Mar 27;20(3):e0014160. doi: 10.1371/journal.pntd.0014160 (PMC13048497; doi:10.1371/journal.pntd.0014160)
Supplement: Attachment — Submitted filename: PNTD-D-25-01961R1 Academic Editor Input.docx [file pntd.0014160.s018.docx]

**ACADEMIC EDITOR’S COMMENTS ON PNTD-D-25-01961R1 (CORDER *ET AL.*)**

1. Line (L) 60: Substitute “PQ” for “primaquine”.

2. L 89 & L 105 & L 339 & L 349: Change “site” to “area” in all four places. And check the supplementary documents in this respect.

3. L 96: Change “1,862 had DNA samples available” to “DNA samples were available for 1,862”.

4. L 102: Consider inserting a full stop (period) after “surface” in this long sentence. Then start a new sentence by inserting “This is” in front of “because” in that line.

5. L 103: Consider inserting a comma after “[20]” and substituting “which” for the word “and” that currently appears behind “[20]”.

6. L 104: Consider inserting “the” between “for” and “*ACKR1*”.

7. L 106: Consider altering “they lacked” to “we lacked”.

8. L 108: Insert the apparently missing closing bracket after “mutation”.

9. L 108: Consider substituting “remained” for “were retained”.

10. L 110: “counted” could potentially be substituted for “enumerated” as a change from the “enumerated” that appears in line 92. Otherwise, leave this as “enumerated”.

11. L 122: Insert “to” between “equal” and “or”.

12. L 147: At the end of the line, delete the “a” in front of “*P. vivax*”.

13. L 157 & L 158: Check the bracketing in these two lines. Is a closing bracket missing?

14. L 232: Does “risk” fit in there in this particular instance (read the sentence carefully)? Should it be deleted or not? If so, should it simply be deleted or replaced by “possibility”?

15. L 248: Change “proxy of” to “proxy for”.

16. L 316: Substitute “varying” for “variable”.

17. L 350: Delete “they lacked” and insert “was not available” at the end of the line, after “information”.

18. L 351: At the end of the line, substitute “remained” for “were retained”.

19. L 353: The style in which dates are set out here (e.g., “01 January 2014”) is not the same as in line 361, for example, where the date appears as “January 1, 2014”. I happen to prefer the former style. Whether or not the journal uses a particular style aside, introduce uniformity in the whole manuscript.

20. L 353: Correct “2028” to “2018”.

21. L 361: Insert the word “and” between the dates, deleting the comma after “2014”.

22. L 361: Correct “2028” to “2018”.

23. L 376: Alter “variable” to “variables”.

24. L 384: Insert a comma after “resources”.

25. L 417 to 419: “We thank the Program …” appears here in the “Financial disclosure” section. That sentence should be moved to the “Acknowledgements” section.

26. L 428: Change “979–990” to “979–90”.

27. L 463: Italicize “Plasmodium vivax”.

28. L 508: Delete the space after “86” as well as “ciae482” so that the full stop after it comes immediately behind “86” (i.e., “… 86.”).

29. L 519: At the end of the line, use a lower case “a” in “an” after “women:”. In other words, change “An” to “an”.

30. L 523: Remove the space between “2026” and the semicolon after it (close up the gap).

MILES B. MARKUS (Academic Editor)
